# Co-expression networks in Chlamydomonas reveal significant rhythmicity in batch cultures and empower gene function discovery

**DOI:** 10.1093/plcell/koab042

**Published:** 2021-02-02

**Authors:** Patrice A Salomé, Sabeeha S Merchant

**Affiliations:** Department of Chemistry and Biochemistry, University of California—Los Angeles, Los Angeles California 90095; Department of Chemistry and Biochemistry, University of California—Los Angeles, Los Angeles California 90095; Departments of Molecular and Cell Biology and Plant and Microbial Biology, University of California-Berkeley, Berkeley, California 94720 and Environmental Genomics and Systems Biology, Lawrence Berkeley National Laboratory, Berkeley, CA 94720

## Abstract

The unicellular green alga *Chlamydomonas reinhardtii* is a choice reference system for the study of photosynthesis and chloroplast metabolism, cilium assembly and function, lipid and starch metabolism, and metal homeostasis. Despite decades of research, the functions of thousands of genes remain largely unknown, and new approaches are needed to categorically assign genes to cellular pathways. Growing collections of transcriptome and proteome data now allow a systematic approach based on integrative co-expression analysis. We used a dataset comprising 518 deep transcriptome samples derived from 58 independent experiments to identify potential co-expression relationships between genes. We visualized co-expression potential with the R package *corrplot*, to easily assess co-expression and anti-correlation between genes. We extracted several hundred high-confidence genes at the intersection of multiple curated lists involved in cilia, cell division, and photosynthesis, illustrating the power of our method. Surprisingly, Chlamydomonas experiments retained a significant rhythmic component across the transcriptome, suggesting an underappreciated variable during sample collection, even in samples collected in constant light. Our results therefore document substantial residual synchronization in batch cultures, contrary to assumptions of asynchrony. We provide step-by-step protocols for the analysis of co-expression across transcriptome data sets from Chlamydomonas and other species to help foster gene function discovery.

## Teaser

We reveal co-expression potential between Chlamydomonas genes and describe strong synchronization of cells grown in batch cultures as a possible source of underappreciated variation.

##  

**Figure:**
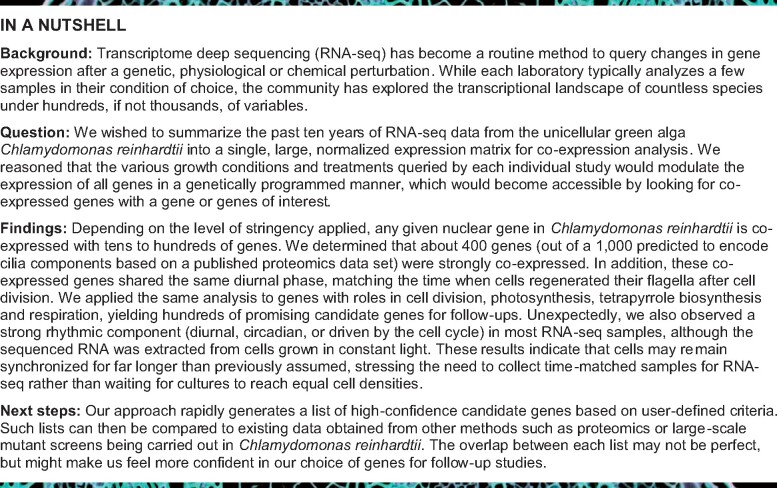


## Introduction

Discovering the functions of genes has driven biology for over a century, using a multitude of tools to determine the factors associated with a given cellular process. In the green unicellular alga Chlamydomonas (*Chlamydomonas reinhardtii*), mutant screens have advanced our understanding in fields such as photosynthesis, metabolism, cell division, and cilium function (Lewin, 1954; Levine, 1960; Ebersold et al., 1962; Levine and Goodenough, 1970; Girard et al., 1980; Erickson et al., 1986; Choquet et al., 1988; Diener et al., 1990; Smith and Lefebvre, 1996; Fleischmann et al., 1999; Depège et al., 2003; Kathir et al., 2003; Rymarquis et al., 2005; Dutcher et al., 2012; Tulin and Cross, 2014; Dent et al., 2015; Li et al., 2019). While the cloning of the causal loci can be painstaking and hindered by our tendency as scientists to guess wrong (Baxter, 2020), Chlamydomonas mutants are amenable to transgenic rescue with large fragments of cloned genomic DNA (Kindle, 1990; Purton and Rochaix, 1994; Zhang et al., 1994), partially circumventing these limitations.

With the advent of sequencing technologies, more holistic and global approaches have been embraced, such as shotgun proteomics analyses of entire organelles or cellular fractions. For instance, the complement of cilia proteins has been determined, aided by the relative ease with which Chlamydomonas cilia can be purified (Pazour et al., 2005). Of the ∼1,000 proteins identified as being part of the cilium, some are likely to represent contaminants during purification or correspond to sticky proteins. Likewise, thousands of genes encode proteins that localize to the chloroplast, where they will participate in various metabolic pathways and photosynthesis. A fraction of these genes is essential for survival, but the majority will have little to no phenotype under laboratory growth conditions when inactivated. In each case, how to prioritize which protein to characterize next is always difficult. One useful approach is to use multiple complementary data types and sources to inform the choice, and integration of genome-scale transcriptome data for a guilt by association perspective can be powerful (Usadel et al., 2009; Baxter, 2020). Expression profiling by microarrays, and later by deep sequencing of the transcriptome (RNA-seq) now provide easy access to the changes of the transcriptome in response to genetic or environmental perturbations. In Chlamydomonas alone, RNA-seq analysis has empowered hypothesis generation by providing a detailed picture of the changes in gene expression in response to light (Xiang et al., 2001; Zhu et al., 2008; Wittkopp et al., 2017), CO_2_ (Fukuzawa et al., 2001; Xiang et al., 2001; Brueggeman et al., 2012; Fang et al., 2012), and stress (Urzica et al., 2012a; Wakao et al., 2014; Blaby et al., 2015; Blaby-Haas et al., 2016), as well as nutritional deficiencies such as nitrogen or iron (González-Ballester et al., 2010; Miller et al., 2010; Castruita et al., 2011; Dudley Page et al., 2012; Urzica et al., 2012b; Blaby et al., 2013; Schmollinger et al., 2014; Kajikawa et al., 2015; Ngan et al., 2015). RNA-seq data have largely been analyzed in a contrasting mode, that is, by comparing the wild type to the mutant, or between untreated and treated cultures, not only in Chlamydomonas but also in other systems. Algal cultures that are sampled for subsequent RNA-seq analysis are generally grown in constant light, with the assumption that, even though individual cells will exhibit circadian and cell cycle-related rhythms, the culture as a whole will be asynchronous. We recently observed significant residual rhythmicity in bulk Chlamydomonas cultures grown in constant light when performing single cell RNA-seq (scRNA-seq), calling this assumption into question (Ma et al., 2021). A rhythmic component during transcriptome analysis can generate false positive (and false negative) associations: in Arabidopsis (*Arabidopsis thaliana*), samples collected 30 min apart from seedlings entrained to light–dark cycles can exhibit differential expression of hundreds of genes that can be explained by the progression of rhythmic gene expression rather than true differential expression (Hsu and Harmer, 2012). Whether the algal circadian clock has any noticeable effect on gene expression profiles of cultures grown in constant light is unknown.

The analysis and synthesis of multiple transcriptome studies is thus critical to covering sufficient experimental conditions to maximize the detection of each transcript under at least one condition, especially when a growth treatment has no available expression data set. Several pipelines have been implemented that combine transcriptomics datasets to build gene regulatory networks and assign gene function (Aoki et al., 2016; Romero-Campero et al., 2016; Nguyen et al., 2019), based on the premise that genes involved in a similar process will be co-expressed, in particular if their encoded proteins physically interact (Ge et al., 2001; Simonis et al., 2004; Komurov and White, 2007; Zhu et al., 2008). However, negative correlations are not generally considered, as one cannot generate anti-correlation networks. We wished to develop a simple alternative to current online-based search tools that can be run on a laptop computer, based on a rich data set from which to extract co-expression and anti-correlation estimates for any gene of interest to facilitate prioritization of candidate genes for classical functional analysis experiments.

We describe here a thorough analysis of the Chlamydomonas transcriptome landscape, based on the analysis of Pearson’s correlation coefficients (PCCs) associated with all nuclear gene pairs using a set of 518 RNA-seq samples from 58 independent experiments. RNA-seq samples from a given experiment were more correlated within the experiment than to samples from any other experiment, even those querying the same variable, indicating the strong environmental sensitivity of Chlamydomonas cultures. We observed frequent co-expression between genes, but also report on anti-correlations, an underappreciated dimension in regulatory networks. We illustrate our approach by revisiting gene lists curated by the Chlamydomonas community and by exploring co-expression modules with visual representation by the R package *corrplot* (Wei and Simko, 2017) and identify high-confidence candidate genes involved in cilia function, photosynthesis, cell division, and the proteasome. Finally, we discovered that the majority of RNA-seq samples exhibits substantial diurnal rhythmicity, even when derived from cells grown in constant light. We provide simple R scripts for data exploration and hope that this resource will be of use to the community, as this approach can be applied to any biological system.

## Results

### Remapping and normalization steps of the Chlamydomonas transcriptome

The analysis of changes in gene expression typically covers a limited number of conditions on selected genotypes to identify treatment-specific modulators of the transcriptome in a given organism. While this approach is powerful, we wished to integrate multiple transcriptome datasets that represent multiple variables in growth conditions and genotypes. To this end, we collected 58 transcriptome deep-sequencing (RNA-seq) datasets, corresponding to 518 samples, generated by the community and by our own laboratory. We remapped all reads to version v5.5 of the Chlamydomonas genome to account for changes in gene models between experiments. We did not attempt to compensate for batch effects or variation in sequencing platforms, which were all Illumina-based but reflected the sequencer in use at that time (Genome Analyzer, Genome Analyzer II, HiSeq1000/2000/2500).

We then assessed the global expression of all 17,741 Chlamydomonas nuclear genes across our set of 518 samples. Most nuclear genes were expressed at levels of 1 fragments per kilobase of transcript per Million mapped reads (FPKM) in most samples, with 59.6% of all genes expressed above 1 FPKM in over 400 of the 518 samples. Only 494 genes (or 2.8% of nuclear genes) never reached an expression value above 1 FPKM (Supplemental Table S1). With a higher threshold for expression, the fraction of expressed nuclear genes decreased: 20.6% of nuclear genes were expressed above a cut-off of 1 FPKM in fewer than 150 samples, but this percentage rose to 69.3% with a cut-off of 10 FPKM, 92.3% with a cut-off of 50 FPKM, and 95.8% with a cut-off of 100 FPKM (Supplemental Table S1). Likewise, the number of genes expressed across at least 501 out of 518 samples dropped from 33.6% (for FPKM > 1) to 5.2% for FPKM > 10, 1.1% for FPKM > 50, and 0.7% for FPKM > 100. Looking at median distributions, each sample had a median gene expression level ranging from 0.6 FPKM to 5.9 FPKM; likewise, each gene showed a median expression level between 0 and 10,134, with an average median of only 30.9. This pattern indicates that most genes are expressed at moderate levels and only in a limited number of conditions (Supplemental Table S1).

We next normalized our RNA-seq data set following the same steps used for the ALCOdb gene co-expression database for microalgae (illustrated in Supplemental Figure S1; Aoki et al., 2016). The final normalization step centered expression estimates to zero, as a *Z*-score normalization would (Supplemental Figure S1,B and Supplemental Figure S1). *RIBOSOMAL PROTEIN GENES* (*RPG*s; Supplemental Data Set S1) illustrated the effect of each normalization step (Supplemental Figure S2).

### Samples from the same experiment show strong positive correlations

**Figure 1:**
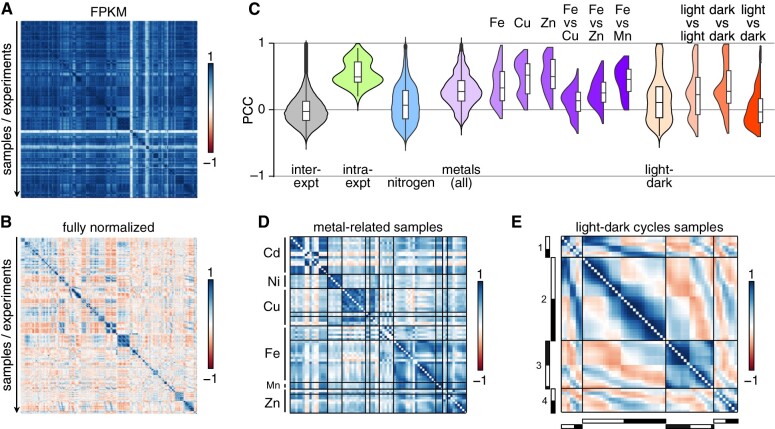
Samples from the same experiment are strongly correlated. **A)**, Correlation matrices between all samples using expression estimates for all 17,741 nuclear genes as FPKM. **B)**, As in panel **A**, but after all normalization steps. In panels **A** and **B**, samples belonging to the same experiment are in consecutive order, and roughly in chronological order. **C)**, Distribution of PCCs between (inter-expt, gray) and within (intra-expt, green) experiments. PCCs for all comparisons between experiments are shown as violin plots and box plots, alongside mean PCCs from all samples within each experiment, samples collected in the context of nitrogen deprivation (blue), PCCs for all metal-related samples (light purple) and specific metals (darker shades of purple), samples collected over a diurnal cycle (light orange), and PCC between subsets of samples (darker shades of orange). Values along the diagonal of the matrix (equal to 1) were discarded prior to plotting. **D)**, Correlation matrix for samples from metal-related experiments, all from the Merchant laboratory, and in which either one micronutrient has been omitted from the growth medium (for deficiency conditions: copper Cu, iron Fe, manganese Mn, and zinc Zn) or a toxic metal was added to observe the effect on homeostasis (cadmium Cd and nickel Ni). **E)**, Correlation matrix of samples collected over a diurnal cycle. The light- and dark-part of each sampling day is indicated on the left and bottom sides of the matrix as white and black bars, respectively. Four time courses are compared here (Panchy et al., 2014; Zones et al., 2015; Strenkert et al., 2019).

This data set allowed us to assess the extent of correlation between samples/experiments (each sample being represented by its unique 17,741 gene expression estimates) or between genes (each gene being characterized by its unique 518 gene expression estimates across all samples). We used the R package *corrplot* to visualize correlations across samples or genes (see Supplemental Figure S3 for details). FPKM values failed to extract a pattern, as most samples were strongly and positively correlated, based on Pearson’s correlation coefficients (PCCs) between samples (Figure 1A; mean PCC = 0.74 ± 0.18). The same held true for log_2_- and quantile-normalized datasets (Supplemental Figure S4; mean PCC of 0.83 ± 0.17). However, mean-centering normalization revealed localized correlation clusters that appeared to be restricted to within each experiment (Figure 1B). Indeed, although the entire correlation matrix had a mean PCC close to zero (0.002 ± 0.226), samples belonging to the same experiment exhibited strong and positive correlations (Figure 1C). Samples from a given experiment (including the reference or control samples) were more related to each other than to any other sample, even when designed to query the same biological question (see, for example, nitrogen deprivation samples, Figure 1C and Supplemental Figure S4,E). Likewise, the laboratory provenance of samples did not explain the extent of relationship between samples: over half of all RNA-seq samples analyzed here have been generated by our laboratory, and yet most failed to exhibit significant correlations outside of each experiment (Supplemental Figure S4,F).

**Figure 2:**
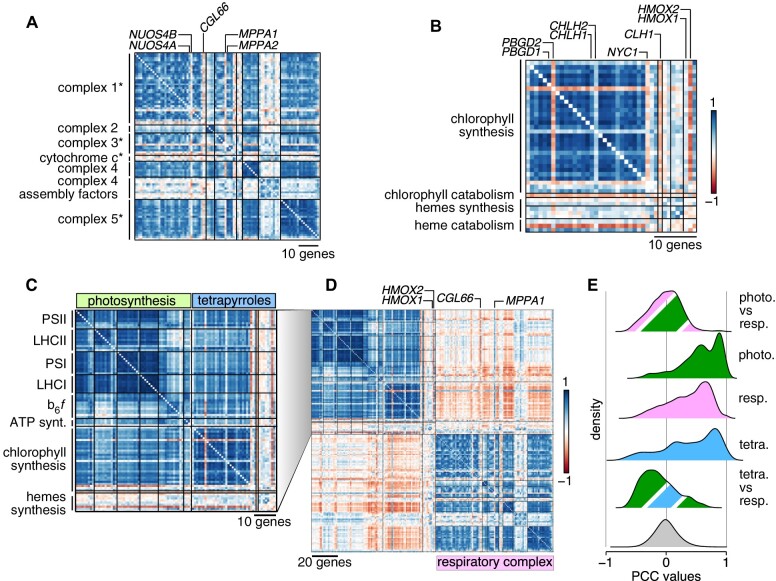
Correlations and anti-correlations between organellar energy producing systems. **A)**, Correlation matrix of nucleus-encoded components of mitochondrial respiratory complexes, in the order defined by Zones et al. (2015). An asterisk after the name of a complex signifies that its dedicated assembly factors (one to two genes outside of complex 4) are shown last, after the complex components. **B)**, Correlation matrix of chlorophyll and hemes biosynthesis genes. Genes have been ordered according to Zones et al. (2015). Pairs of homologous genes are indicated above the correlation matrix. **C)**, Co-expression matrix of photosystem genes (in green) and tetrapyrroles biosynthetic genes (in blue). **D)**, Comparison of co-expression profiles of chloroplast- and mitochrondrion-localized energy production systems. The respiratory complex matrix is redrawn from Supplemental Figure S9.E, Distribution of PCCs between groups of genes. The gray distribution is the genome-wide distribution of all PCCs between all gene pairs. photo., photosynthesis; tetra., tetrapyrroles; resp., respiration.

Two sets of experiments deviated from the general trend: experiments that were 1) metal-related (Figure 1D) or 2) that spanned a diurnal cycle (Figure 1E). Positive correlations largely segregated samples collected from cultures lacking a single micronutrient (Cu, Fe, Mn, or Zn) into their targeted deficiency. Based on correlations across samples, Fe-deficient cultures were slightly more similar to Zn- and Mn-deficient cultures than they were to Cu-deficient cultures (Figure 1C), as expected. These observations support the hypothesis that these three metals (Fe, Zn, and Mn) are transported by partially overlapping sets of transporters and involve partially shared regulon components (Merchant et al., 2006; Malasarn et al., 2013; Hong-Hermesdorf et al., 2014; Tsednee et al., 2019).

The correlation matrix between diurnal samples was striking: we observed the highest degree of positive correlation between samples that were temporally close to one another within and across diurnal experiments (Figure 2E). At a slightly broader scale, samples collected during the day were generally positively correlated, again within and across diurnal experiments, although the extent of correlation was stronger between samples from the same experiment. The same observation held true when comparing samples collected during the night part of the diurnal cycle. Finally, samples collected during the day were negatively correlated with samples collected at night, both within and across experiments (Figure 1E). In all diurnal samples, over 80% of nuclear genes exhibited a rhythmic pattern with phases spanning the entire day (Zones et al., 2015; Strenkert et al., 2019). That diurnal samples can cluster so clearly according to their collection time suggests that the endogenous timing of an unknown sample might be accessible by comparing its correlation profile with that of known diurnal datasets. This approach is similar in concept to the molecular timetable method used to detect sample time from single time-point data (Ueda et al., 2004).

### Co-expression potential in manually curated gene lists

We next turned our attention to correlation between genes to dissect co-expression potential in Chlamydomonas. We calculated PCCs for all gene pairs (157,362,670 pairs, not counting self–self pairs); they followed a normal distribution (Kolmogorov–Smirnov test statistic *D* = 0.019, *P*-value < 2.2 × 10^−16^), indicating that most gene pairs are not co-expressed (Supplemental Figure S5,A).

Hierarchical clustering suggested that sets of genes displayed highly similar expression behaviors (Supplemental Figure S5,B and C). A cursory exploration of our data set indicated that we recapitulate known patterns of co-expression (Supplemental Data Set S2). For instance, the genes *LHCSR3.1* and *LHCSR3.2* are induced in response to high light, as are the genes *PSBS1* and *PSBS2*: we saw the same pattern illustrated in our data set, although most samples were not collected under high light conditions (Supplemental Figure S6,A). Likewise, we characterized the correlation pattern in the expression of heat shock genes *HSP70* and *HSP90* and the plastid chaperonin genes *CPN60*: their expression was largely correlated, with stronger co-expression between members of the same gene family (Supplemental Figure S6,B). Genes involved in nitrogen uptake and assimilation similarly showed strong co-expression, with some known exceptions; for example, the ammonium transporters *AMT6* and *AMT7* were anti-correlated with other transporters *AMT1*, *ATM4*, and *AMT5* (Supplemental Figure S6,C), which is consistent with their transcriptional repression in response to nitrogen deprivation, in contrast to the other transporters (Schmollinger et al., 2014).

Based on these encouraging observations, we followed a three-pronged approach to test for co-expression and identify co-expressed genes. First, we determined the extent of co-expression and anti-correlation in gene lists manually curated from the community. Second, we defined the co-expression cohort associated with a given nuclear gene. Third, we identified co-expression modules. Both latter approaches entailed calculating the mutual rank (MR) associated with each gene pair (Obayashi and Kinoshita, 2009; Aoki et al., 2016; Wisecaver et al., 2017). We then turned MRs into edge weights as a measure of the connection between co-expressed genes (or nodes) for the construction of five MR-based co-expression networks with decreasing decay rates, denoted N1–N5. During this process, we identified all genes that were co-expressed with each individual nuclear gene (Supplemental Files S2–S4 for networks N1–N3) and their anti-correlated cohorts, by inverting the rank order (Supplemental Files S5–S7). Each gene was at the center of a co-expression cohort with a clustering coefficient of zero (Supplemental Table S2). Under the most stringent criteria for co-expression, a Chlamydomonas gene was co-expressed with 1–68 genes, with a mean cohort size of 17 genes. Relaxing the stringency imposed on co-expressed genes increased the mean size of cohorts to 36 (N2 networks) and 98 genes (N3 networks) (Supplemental Table S2).

As a proof of concept, we turned to gene lists compiled by the community. These lists comprised genes that participate in the same biological function or pathway, but information about their co-expression potential is incomplete. In addition, most co-expression analyses focus on positive correlations as the core criterion for the identification of co-expressed groups, and largely ignore anti-correlated genes. Here, we tested 1) whether genes from a list were co-expressed and 2) whether the expression profile of any gene within the lists was anti-correlated with others.

**Figure 3:**
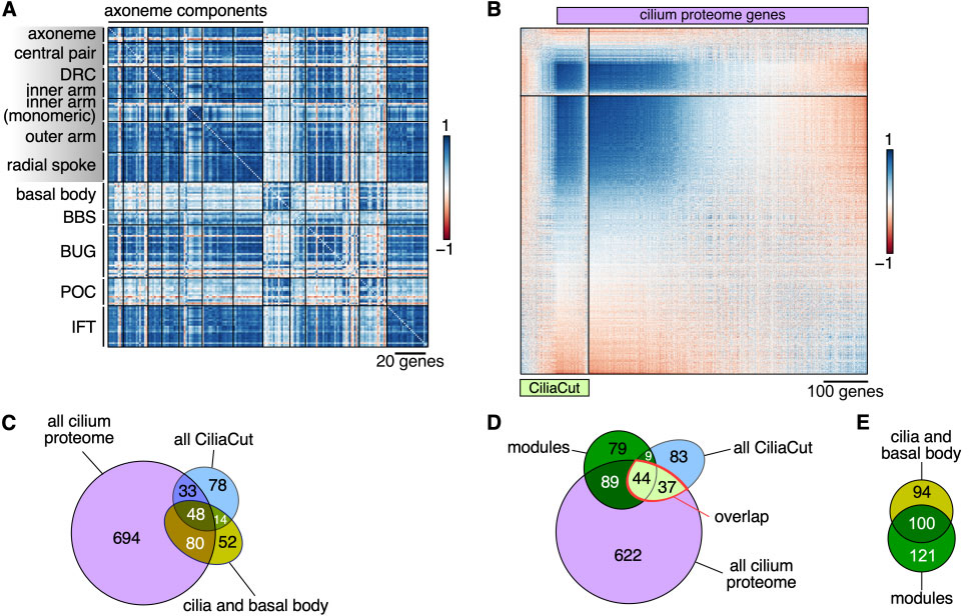
Confirmation of high-confidence cilium proteins based on co-expression of their encoding genes. **A)**, Correlation matrix of structural constituents of the Chlamydomonas cilium, in the order defined by Zones et al. (2015). DRC, dynein regulatory complex; BBS, Bardet–Biedl syndrome protein complex; BUG, basal body upregulated after deflagellation; POC, proteome of centriole; IFT, intra-flagellar transport. **B)**, Correlation matrix between genes belonging to CiliaCut (green) or encoding components identified in the cilium proteome (light purple; Pazour et al., 2005). The genes within each subset were subjected to hierarchical clustering (FPC method in *corrplot*). **C)**, Venn diagram of the overlap between genes encoding putative components of the cilium proteome, CiliaCut, and the cilia and basal body. Note that the gene lists do not reflect co-expression here. **D)**, Venn diagram of the overlap between genes encoding putative components of the cilium proteome, CiliaCut, and genes belonging to cilia-related co-expression modules (listed in Supplemental Table S3). E, Venn diagram of the overlap between genes encoding putative components of the cilia and basal body and genes belonging to cilia-related co-expression modules.

**Figure 4:**
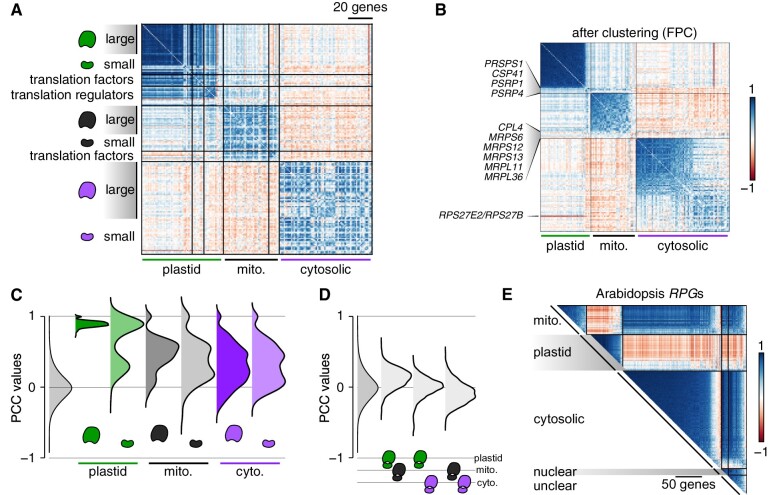
Co-expression between *RPG*s reflects the final location of the corresponding ribosomal proteins. **A)**, Correlation matrix between *RPG*s (Supplemental Data Set S1) and their translation regulators, sorted by the subcellular localization of their encoded proteins. For each set of *RPG*s and their regulators, we followed the same gene order defined by Zones et al. (2015). **B)**, Correlation matrix restricted to *RPG*s. Each set of *RPG*s was subjected to hierarchical clustering (FPC method in *corrplot*) to single out non-co-expressed genes. **C)**, Distribution of PCCs between *RPG* gene pairs encoding large or small ribosome subunits. The gray distribution indicates the PCC distribution of all gene pairs for the Chlamydomonas genome. **D)**, Distribution of PCCs for gene pairs belonging to distinct *RPG* groups. **E)**, Correlation matrix for 357 *RPG*s (Supplemental Data Set S5) using the fully normalized dataset derived from Arabidopsis microarray experiments (Supplemental Data Set S6). “Nuclear” and “unclear” denote *RPG*s whose encoded proteins are predicted to localize to the nucleus or lack a clear localization, respectively.

**Figure 5:**
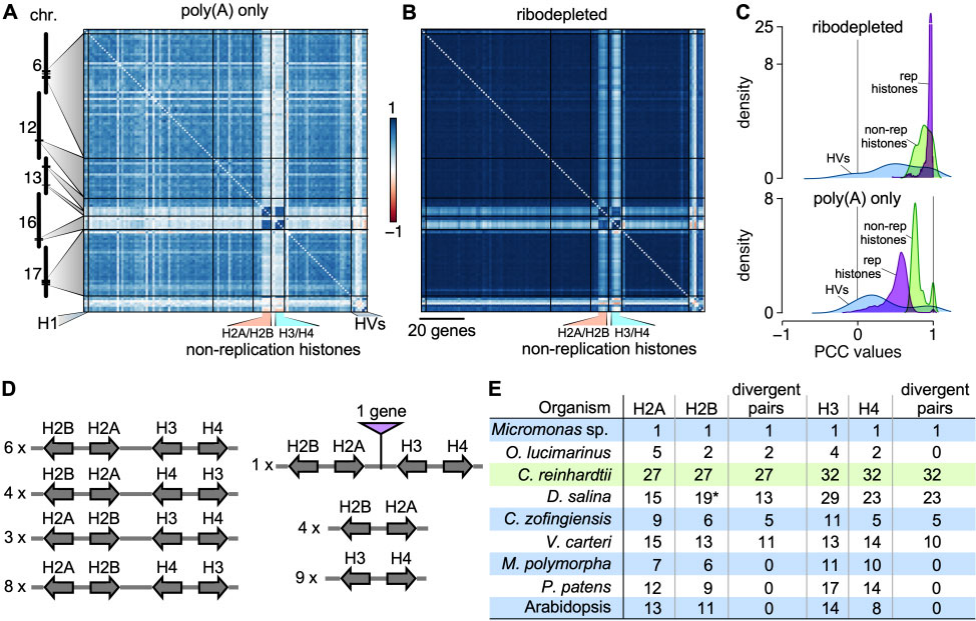
Correlations between Chlamydomonas histone genes. **A)**, Correlation matrix among Chlamydomonas histone genes, ordered according to their genomic coordinates, using RNA-seq data derived from poly(A)-selected samples. **B)**, Same as (**A**), using RNA-seq data derived from ribodepleted samples. Histone genes that are not regulated by the cell cycle are indicated as “non-replication histones.” H1, histone H1 genes; HVs, histone variants. **C)**, Distribution of PCCs for classes of histones genes shown in (**A**) and (**B**). Histone variants (HVs) are shown in light blue, replication-associated histones in purple, and non-replication histones in light green. **D)**, Global clustering of histone genes in Chlamydomonas. All histone genes occur as divergent pairs and are oftentimes grouped as one representative of each major histone type (H2A, H2B, H3, and H4). The number to the left gives the number of instances of the given arrangement in the Chlamydomonas genome. **E)**, Comparison of histone gene clustering in selected photosynthetic organisms. *O. lucimarinus*, *Ostreococcus lucimarinus*; *D. salina*, *Dunaliella salina*; *V. carteri*, *Volvox carteri*; *C. zofingiensis*, *Chromochloris zofingiensis*; *M. polymorpha*, *Marchantia polymorpha*; *P. patens*, *Physcomitrium patens*. The asterisk for *Histone H2B* genes in *D. salina* indicates that they are absent from the current annotation, but were identified by TBLASTN against the *D. salina* genome with Chlamydomonas histone H2B protein sequence as query.

Since Chlamydomonas is a premier reference organism for organellar biogenesis and cilia biosynthesis and biology, we determined the co-expression potential of genes encoding components of the mitochondrial respiratory chain (Supplemental Data Set S3), photosystems, and biosynthesis of chlorophyll and hemes (Supplemental Data Set S4 and Figure 2), as well as motile cilia (Supplemental Data Set S5 and Figure 3). We also assessed the co-expression potential of ribosome protein genes (*RPG*s) in Chlamydomonas (Supplemental Data Set S1 and Figure 4), as much early work in Chlamydomonas has described the organellar protein translation machinery in detail (Sager and Hamilton, 1967; Siersma and Chiang, 1971; Ohta et al., 1975; Martin et al., 1976). Finally, we tested co-expression between histone genes in Chlamydomonas (Figure 5).

#### Nucleus-encoded organellar energy systems

Mitochondria and chloroplasts provide energy and reducing power to the cell, although the underlying genes may show distinct expression profiles. Based on previous results (Zones et al., 2015; Strenkert et al., 2019), we expected to observe global co-expression of genes encoding components of the mitochondrial respiratory complex. Indeed, most genes whose products participate in mitochondrial electron transport or oxidative phosphorylation were co-expressed (Figure 2A), although some genes deviated from this pattern. For instance, *CONSERVED IN THE GREEN LINEAGE 66* (*CGL66*, Cre09.g390467) was negatively correlated with other complex 1 genes, suggesting that it may not belong to this complex, or functions as a negative regulator. Proteins encoded by two related genes provided an example of potential sub-functionalization: *NUOS4B* (Cre16.g681700, from complex 1) and *MITOCHONDRIAL PROCESSING PEPTIDASE ALPHA SUBUNIT* (*MPPA1*, Cre17.g722800, from complex 3) were not co-expressed with other genes coding for components forming their respective complexes, although the related genes *NUOS4A* and *MPPA2* were (and were also more highly expressed).

Of the genes involved in tetrapyrroles biosynthesis, only those encoding enzymes responsible for chlorophyll biosynthesis appeared to be co-expressed, with the exception of the porphobilinogen deaminase gene *PBGD2* (Cre02.g113850) and the magnesium chelatase subunit H gene *CHLH2* (Cre11.g4776625), although their homologs *PBGD1* and *CHLH1* were (Figure 2B), with *PBGD1* expressed at much higher levels than *PBGD2*. By contrast, heme biosynthetic genes exhibited no co-expression with genes from either photosystem (mean PCC: –0.03 ± 0.23).

All photosynthetic genes were strongly co-expressed (Figure 2B). Although heme and chlorophyll biosynthesis compete for the same pool of precursors, the expression of the genes involved in each pathway was independent (mean PCC: 0.04 ± 0.28). Genes encoding heme-containing enzymes and other cytochromes were however anti-correlated with chlorophyll biosynthetic genes (Figure 2B–D), thereby ensuring that adequate levels of heme be synthesized without reaching toxic levels by coordinating the heme pool with heme binding proteins. The two heme oxygenase genes followed distinct expression behaviors: *HMOX1* was weakly co-expressed with photosystems and other tetrapyrrole biosynthetic genes, whereas *HMOX2* was strongly anti-correlated with them, consistent with the light-dependent repression of this gene (Wittkopp et al., 2017). Furthermore, the *hmox1* mutant is pale-green, a phenotype typical for chlorophyll biosynthesis mutants. Notably, the expression of genes involved in photosynthesis is not affected in the *hmox1* background, which is consistent with the general lack of correlation between *HMOX1* and photosystems (Wittkopp et al., 2017).

Finally, genes encoding proteins that form the mitochondrial respiratory complex were largely anti-correlated with photosynthetic and tetrapyrrole biosynthetic genes (Figure 2D and E). This anti-correlation may partially stem from the distinct temporal separation of the underlying cellular events: high expression during the day for photosynthesis and tetrapyrroles biosynthesis, and high expression in two peaks, one in the middle of the night and a second one during the day for mitochondrial respiration (Zones et al., 2015; Strenkert et al., 2019). That respiratory complex genes are expressed in the middle of the day coincides with the higher respiration rate seen at that time (Strenkert et al., 2019).

#### Cilia

The components of the Chlamydomonas cilia are coordinately transcribed following cell division at night, as cells first resorb their existing flagella prior to division and must synthesize a new pair for a daughter cell in anticipation of dawn and photosynthetic activity (Rosenbaum et al., 1969; Wood et al., 2012; Cross and Umen, 2015). Although most RNA-seq samples were collected from cultures grown in constant light and, presumably, asynchronous, we observed strong co-expression across most genes encoding structural components of the cilia (mean PCC: 0.65 ± 0.18), as well as with components of IntraFlagellar Transport (IFT) particles responsible for the assembly, maintenance, and signaling within cilia (mean PCC: 0.74 ± 0.17) (Figure 3A). Several cilia-related genes did not follow this general trend: they encoded proteins that modify protein function and therefore act at the post-translational level (Flagella Associated Protein 8 [FAP8], a protein phosphatase 2A regulator; enolase, contributing to ATP production within cilia, and a number of chaperones or heat shock proteins [DNJ1, HSP70A]). Other genes that were not co-expressed encoded proteins with cellular roles outside of cilia, for instance HSP70A, actin, and profilin, suggesting that a fraction of the total pool of each protein participates in cilia biogenesis while the bulk carries out functions in the cytosol.

Centriole proteins have been identified by a number of techniques, including mass spectrometry of purified centrioles, co-expression following deflagellation, and comparative genomics (Li et al., 2004; Keller et al., 2005; Keller and Marshall, 2008). Genes encoding most basal body components were indeed co-expressed across all our samples and showed strong co-expression with *PROTEOME OF CENTRIOLE* (*POC*) genes. Both basal body and *POC* genes were however only weakly co-expressed with genes coding for cilia components, as might be expected: the centriole is always present in the cell, whereas cilia form a more dynamic structure (Figure 3A). As previously described, the majority of *BASAL BODY UPREGULATED AFTER DEFLAGELLATION* (*BUG*) genes were more co-expressed with cilia components than with basal body markers (Figure 3A). The co-expression profile of several *BUG* genes (*BUG23*, *BUG24*, *BUG27*) suggested that their function may be instead associated with the centriole proper, as they showed stronger co-expression with basal body genes. We also noted a lack of co-expression between basal body components and *CCT3*, *HSP90A*, *FMO11*, and *PHB1*, all predicted to perform function(s) outside of the centriole (Zones et al., 2015).

Genes encoding components of the Bardet–Biedl syndrome protein complex (BBSome) were only weakly co-expressed (mean PCC: 0.29 ± 0.16) and were not co-expressed with basal body constituents (mean PCC: 0.23 ± 0.16), while moderately with ciliary structures (mean PCC: 0.38 ± 0.23). Our co-expression analysis of cilia and centriole components therefore accurately grouped genes based on function and cellular localization and highlighted those genes with distinct expression profiles. The ability to identify bona fide cilia and centriole components based on co-expression also offered the opportunity to subject larger lists to a similar analysis. The cilium proteome is predicted to comprise close to a thousand proteins based on proteomics analysis (Pazour et al., 2005), although a fraction is likely to correspond to contaminants. Likewise, a comparative genomics approach uncovered around 200 genes encoding proteins conserved between ciliated species and absent in all other species, referred to as “CiliaCut” (Merchant et al., 2007). These two lists overlap only partially, with 81 genes belonging to both. We wondered if co-expression profiling might allow to pull high-confidence cilia components: we measured co-expression in three groups (CiliaCut only; CiliaCut+cilium proteome overlap; cilium proteome only). The resulting correlation matrix is shown in Figure 4B. Genes only included in the CiliaCut set were on average not co-expressed with each other (mean PCC: 0.03 ± 0.24) and consisted of many *MOTILITY* (*MOT*) genes not found in *Caenorhabditis elegans* (which lacks motile cilia) and *SENSORY, STRUCTURAL AND ASSEMBLY* (*SSA*) genes (Merchant et al., 2007). Similarly, about 550 genes only present in the cilium proteome gene list showed no pattern of co-expression, with a mean PCC of 0.01 ± 0.22. In sharp contrast, 76 genes that belonged to both lists were highly co-expressed (mean PCC: 0.63 ± 0.20). Equally highly co-expressed was a set of ∼300 genes whose encoded proteins are only found in the cilium proteome (mean PCC: 0.63 ± 0.15), with many uncharacterized *FLAGELLAR ASSOCIATED PROTEIN* (*FAP*) genes. Together, these two sets comprised over 400 co-expressed genes that are prime candidates for functional dissection (Supplemental Data Set S5).

#### Ribosome protein genes

Nucleus-encoded *RPG*s code for proteins with three cellular destinations. The co-expression pattern observed between *RPG*s largely reflected the organelle in which their encoded subunits will function (Figure 4A). Plastid *RPG*s exhibited the strongest degree of co-expression (mean PCC = 0.88 ± 0.06). The sole exceptions were the *PLASTID SPECIFIC RPG*s *PSRP1* and *PSRP4*, which are among the lowest expressed genes encoding small subunits proteins, and the gene encoding the Chloroplast Stem-loop binding Protein of 41 kD, CSP41 (mean PCC = 0.27 ± 0.09) (Figure 4B). Neither PSRP1 or CSP41 are thought to be plastid ribosomal proteins, but both participate in efficient translation, either by inducing conformational changes within the ribosome (PSRP1, Sharma et al., 2010) or by stabilizing target plastid RNAs (CSP41, Qi et al., 2012). Large and small plastid ribosomal subunits were co-expressed equally strongly (*PRPL*s: 0.89 ± 0.04; *PRPS*s: 0.86 ± 0.09 excluding *PSRP1* and *PSRP4*) (Figure 4C). Plastid translation factors also displayed a high degree of co-expression with one another (mean PCC: 0.52 ± 0.18) and with plastid *RPG*s (mean PCC: 0.59 ± 0.20). Co-expression between chloroplast translation regulators defined three sub-groups: one group that was highly co-expressed with plastid *RPGs* (11 genes), one group that was not co-expressed (four genes: *RNA-BINDING PROTEIN 38 RB38*, *ACETATE REQUIRING 115 AC115*, *BUNDLE SHEATH DEFECTIVE2 BSD2*, and *CHLOROPLAST RHODANESE-LIKE TRANSLATION CRLT*), and a single weakly anti-correlated gene with all plastid *RPG*s, the translation regulator *TBA1* (*translational affector of* psbA; mean PCC against *RPG*s: –0.35 ± 0.19).

The co-expression of *RPG*s encoding proteins destined for the mitochondrion or cytosol was less pronounced, but similar between large and small subunits *RPG*s (Figure 4C). For both compartments, correlation coefficients between *RPG*s followed a bimodal distribution, with a fraction of PCCs around zero. For mitochondrial *RPG*s, high expression levels appeared to come at the cost of lower PCCs, whereas the opposite was true for cytosolic *RPG*s. Mitochondrial *RPG*s tended to be weakly co-expressed with plastid *RPG*s (mean PCC: 0.13 ± 0.14) while anti-correlated with cytosolic *RPGs* (mean PCC: –0.08 ± 0.15) (Figure 4D). There was no clear correlation between the expression of most plastid and cytosolic *RPG*s (mean PCC: −0.0006 ± 0.14) (Figure 4D). As the single exception, the cytosolic *RPG RPS27E2/RPS27B*, which is generally expressed at much lower levels than all other cytosolic *RPG*s, stood out with a pronounced anti-correlation with plastid *RPGs* (mean PCC: –0.54 ± 0.05) (Figure 4B). Nitrogen deficiency results in a sharp increase in *RPS27E2* expression, concomitant with a global arrest in plastid translation until more auspicious conditions return (Schmollinger et al., 2014; Kajikawa et al., 2015), which may explain the pattern observed here.

Given the strong correlation between sets of *RPG*s in Chlamydomonas, we tested whether Arabidopsis *RPG*s might exhibit a similar pattern next. Accordingly, we subjected microarray data sets downloaded from AtGenExpress to the same normalization steps described above. The Arabidopsis genome contains 429 *RPG*s (Sormani et al., 2011); of those, 357 were represented by a probe on the ATH1 Affymetrix microarray and were predicted to encode ribosomal proteins localizing to the cytosol (184), mitochondria (55), chloroplasts (69), or with an unclear localization (49, including 13 with a predicted nuclear location) (Supplemental Data Set S6). We extracted their normalized expression values from Supplemental File S8, calculated the associated PCCs and reordered each *RPG* subgroup as a function of their clustering with the first principle component (FPC) method in *corrplot*. The resulting correlation matrix was reminiscent of that seen with Chlamydomonas *RPGs*: indeed, each organellar *RPG* set was co-expressed, except for 17 cytosolic *RPG*s with low to negative PCCs (Figure 4E). Plastid *RPG*s were globally anti-correlated with mitochondrion and cytosolic *RPG*s, which would be consistent with a temporal allocation of amino acids to each group of ribosomes, highly abundant proteins that impose high nitrogen demands on the cell. In addition, cytosolic *RPG*s showed a stronger correlation pattern with other cytosolic *RPG*s than they did with mitochondrion *RPG*s, providing a possible signature for the final subcellular location of the encoded proteins. Finally, *RPG*s encoding proteins with an unclear localization appeared to be highly correlated with mitochondrion and cytosolic *RPG*s, but not with plastid *RPG*s, thus making it unlikely that this *RPG* subset would encode ribosomal proteins with plastid localization (Figure 4E).

#### Histones

Turning to Chlamydomonas genes encoding DNA-binding proteins, we took a closer look as histone genes (Supplemental Data Set S7), most of which are coordinately expressed with a peak in expression shortly before cell division as non-polyadenylated transcripts (Zones et al., 2015; Strenkert et al., 2019). A small group of histone genes also remain constantly expressed over the diurnal cycle and are termed “non-replication” (or emergency) histones; their transcripts are polyadenylated. We therefore separated samples from ribodepleted sequencing libraries from all others, and re-ran the normalization steps on both sets of samples (polyA-selected and ribodepleted). Non-replication histone genes were highly co-expressed in both subsets, with a mean PCC of 0.77 ± 0.05 across polyA-selected samples (Figure 5A and C) and a mean PCC of 0.86 ± 0.08 across ribodepleted samples (Figure 5B and C). Although replication histones showed high co-expression in the same data set, with a mean PCC of 0.51 ± 0.16, we hypothesized that much of this pattern is an artifact of the normalization to the mean, which will overinflate their variation in expression. However, replication histones were clearly globally co-expressed, as demonstrated by their high (0.96 ± 0.05) mean PCC when restricting the data set to ribodepleted samples (Figure 5B and C). Histone variants showed weaker and more variable correlation, with mean PCCs of 0.24 ± 0.26 across polyA-selected samples and 0.44 ± 0.32 in ribodepleted samples (Figure 5A–C).

While assembling the gene list for histones, we noticed that all histone genes were arranged as divergent gene pairs: all histone H2A and H2B genes were present as divergent pairs, and all histone H3 genes occurred as a divergent partner to a histone H4 gene. In many cases, each major histone class was represented in a four-gene cluster, corresponding to 84 (out of 117) histone genes (Figure 5D and E). To determine how widespread this histone arrangement might be, we surveyed the histone gene family in the algae *Volvox carteri*, *Chromochloris zofingiensis*, *Dunaliella salina*, *Ostreococcus lucimarinus*, and *Micromononas* sp.: in all cases, most histone genes occurred as divergent gene pairs (Figure 5E and  Supplemental Data Set S8). For example, in *Micromonas* sp., the four histone genes were arranged as two divergent pairs, with H2A and H2B belonging to one pair, and H3 and H4 found in the second pair. Likewise, most histone genes from *C*. *zofingiensis*, *D. salina*, and *V. carteri* grouped in divergent pairs. By contrast, the genomes of the liverwort *Marchantia polymorpha*, the moss *Physcomitrium* (*Physcomitrella*) *patens*, and the land plant Arabidopsis showed no such arrangement (Figure 5E), hinting at the complex evolutionary history of the histone gene family.

### Co-expression modules

We next used our co-expression cohorts and associated edge weights as input for the graph-clustering Cytoscape plugin ClusterONE (Nepusz et al., 2012), resulting in the identification of 616 co-expression modules for network N1, 248 modules for network N2, and 117 modules for network N3 (Supplemental Figure S7 and Supplemental Table S2). We restricted our efforts to the N3 network as a good compromise between larger module sizes and significant GO enrichment within modules. Out of 117 N3 modules, we grouped 37 modules into 8 functional groups based on their significant enrichment in biological processes: transcription, translation, ribosome biogenesis, protein degradation, DNA replication, transport, photosynthesis, and flagella biogenesis and function (Supplemental Table S3 and Supplemental File S9). A single module defined a ninth group associated with response to phytohormones, specifically cytokinin, whose signaling cascade is incomplete in the microalga (Lu and Xu, 2015). These categories were not surprising: they broadly mapped to conserved cellular functions, or to processes where Chlamydomonas is a premier model organism for their study.

To obtain genes that are co-expressed with a list of interest, we separately used manually curated gene lists as baits to extract their co-expressed genes from the N1, N2, and N3 networks. As stringency decreases from the N1 to the N3 networks, the number of selected genes increased, but the resulting lists were nested. Co-expression cohorts associated with gene lists expanded the number of potentially informative genes 2–20 fold, with an average increase of 10-fold (Supplemental Figure S8). Using genes from co-expression modules as baits, we thus identified their associated co-expressed cohorts and determined the extent of overlap with other user-defined lists (as illustrated in Figure 3C) to obtain high-confidence genes. We also established the timing of peak expression over the diurnal cycle for each module, group, and co-expressed cohorts, using the diurnal phase of all genes considered rhythmic in two diurnal datasets (Supplemental Figure S9; Zones et al., 2015; Strenkert et al., 2019).

#### Cell division modules

**Figure 6:**
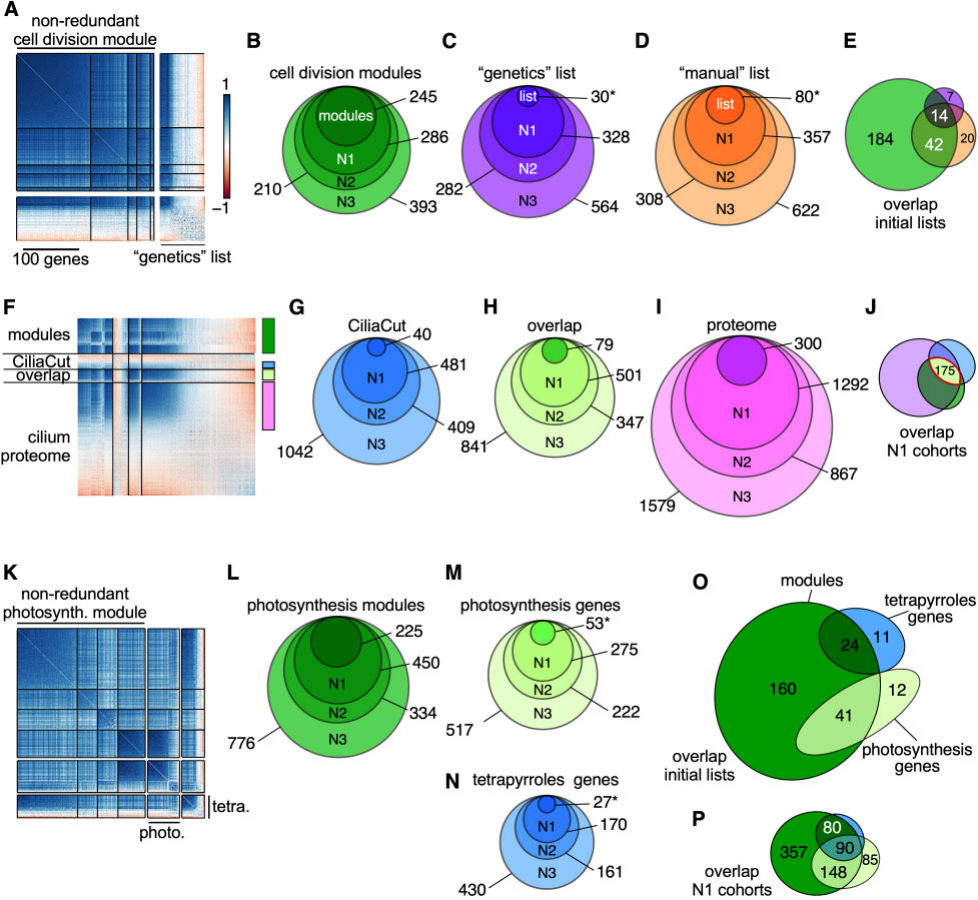
Core cell division genes are coordinately and highly co-expressed. **A)**, Correlation matrix of non-redundant cell division modules and correlation matrix of genes whose loss of function leads to cell division defects (Tulin and Cross, 2014; Breker et al., 2018). Genes within each set were ordered according to hierarchical clustering using the FPC method in *corrplot*. **B–D)**, Co-expressed cohorts, shown as nested Venn diagrams, associated with genes from the cell division modules (**B**), the genetics list (**C**), or genes involved in DNA replication and chromosome segregation (manual list) (**D**) from networks N1–N3. **E)**, Overlap between original gene lists related to cell division (modules, genetics, and manual lists). **F)**, Correlation matrix of non-redundant cilia modules (modules) and genes belonging to CiliaCut only (CiliaCut), the cilium proteome and shared genes between CiliaCut and the cilium proteome (overlap). The color bars on the right refer to the color scheme used for co-expression cohorts in **G–J**. **G–I)**, Co-expressed cohorts, shown as nested Venn diagrams, associated with genes from CiliaCut (**G**), the overlap between CiliaCut and the cilium proteome (**H**), and the cilium proteome (**I**) from networks N1–N3. **J)**, Overlap between N1 cohorts associated with each initial gene list (CiliaCut, overlap, and cilium proteome). **K)**, Correlation matrix of non-redundant photosynthesis modules, photosynthesis-related genes, and tetrapyrrole biosynthesis-related genes. **L–N)**, Co-expressed cohorts, shown as nested Venn diagrams, associated with genes from the photosynthesis modules (**L**), photosynthesis-related genes (**M**), and tetrapyrrole biosynthesis-related genes (**N**) from networks N1–N3. **O)**, Overlap between initial gene lists. **P)**, Overlap between N1 cohorts associated with photosynthesis and tetrapyrrole biosynthesis. In panels **C**, **D**, **M**, and **N**, the asterisk indicates that the gene list was restricted to highly co-expressed genes, based on FPC clustering of the data.

Five modules involved in cell division and DNA replication comprised a non-redundant set of 245 genes (Figure 6A), with 88 genes with an acronym and 157 with no prior functional knowledge. Using guilt by association, we propose that these non-annotated genes play a role in some aspect of cell division. Only 19 out of the 245 genes overlapped with 79 genes identified by forward genetic screens for defects in cell cycle progression; this overlap was limited to the highly co-expressed genes within both sets (Figure 4A;  Tulin and Cross, 2014; Breker et al., 2018). We then determined the co-expression cohorts associated with each gene list and assessed their overlap. By definition, all genes within our modules are highly inter-connected, but they also exhibited co-expression with ∼400 additional genes that define a larger cohort with presumptive function in cell division (Figure 6B). Similarly, hundreds of genes showed strong co-expression with the 30 co-expressed genes from the genetics list (Figure 6C). Finally, we defined a third list comprising genes critical for DNA replication, chromosome segregation, and cell division proper, for which we determined the co-expression cohorts (Figure 6D and Supplemental Data Set S9). Notably, although the initial gene lists were distinct (Figure 6E), their cohorts shared more genes as network stringency decreased, suggesting that the intersection of co-expression cohorts converged on a common set of genes.

#### Proteasome-dependent protein degradation

Two modules shared a function in protein degradation. They largely overlapped and defined a set of 96 genes that included all but two of the 26S proteasome subunit genes. Most genes encoding subunits of the 26S proteasome were highly co-expressed (mean PCC: 0.67 + 0.13). *CSN2* and *CSN6* were however not part of the protein degradation modules; they exhibited the weakest co-expression profile with other 26S proteasome subunit genes, although clearly still quite high (*CSN2* mean PCC: 0.54 ± 0.15; *CSN6* mean PCC: 0.53 ± 0.06) (Supplemental Figure S10,A). The Chlamydomonas ortholog for the E3 ubiquitin ligase CONSTITUTIVE PHOTOMORPHOGENIC 1 (COP1), Cre13.g602700 (currently annotated as SPA1, Gabilly et al., 2019), showed no co-expression with the 26S proteasome (mean PCC: –0.09 ± 0.10), consistent with a role as a regulatory component of the proteasome. We observed the same absence of co-expression in Arabidopsis between *COP1* and the remaining subunits of the proteasome, indicating a conserved mode of control from unicellular algae to land plants.

Proteasome-dependent proteolytic degradation entails the addition of ubiquitin onto the protein targeted for removal by the concerted action of E1 ubiquitin-activating enzymes, E2 ubiquitin-conjugating enzymes, and E3 ubiquitin ligases. The Chlamydomonas genome contains 13 genes for ubiquitin, three genes encoding potential E1 enzymes (Cre09.g386400, Cre06.g296983, and Cre12.g491500) and 17 genes coding for E2 enzymes. We did not compile a list of all E3 ubiquitin ligase genes, as they form large gene families, and respond to various different signaling pathways. Our protein degradation modules only incorporated a single gene each for ubiquitin (*UBQ2*), E1 activating enzyme (Cre12.g491500, annotated as *UBA2*), and E2 conjugating enzyme (*UBC21*, although it was the second lowest-expressed *UBC* gene in our dataset; Supplemental Figure S10,A). No other ubiquitin gene displayed a co-expression pattern with our protein degradation modules. By contrast, both remaining E1 enzyme genes (Cre09.g386400 and Cre06.g296983) were highly co-expressed with genes from our protein degradation modules. Likewise, we identified a subset of genes encoding E2 conjugating enzymes that were co-expressed with 26S proteasome subunit genes: *UBC3* (Cre03.g167000), *UBC9* (Cre16.g693700, also the most highly expressed *UBC* gene), and *UBC13* (Cre01.g046850) and present in the co-expression cohort linked to our modules. In addition, the gene *UBC22* (Cre12.g515450) appeared anti-correlated with other 26S proteasome subunit genes, hinting at a previously unexpected level of control.

We used the 96 genes that formed the protein degradation modules as baits to identify their co-expressed cohorts in each of our three most stringent networks (N1–N3). Via guilt by association prediction, we thus assigned a potential function in protein degradation for 350–760 genes in addition to those already found within our modules (Supplemental Figure S10,B and Supplemental Data Set S10).

#### Cilia modules

Four modules were associated with GO terms with a function in cilia assembly or intraciliary transport. They also demonstrated partial overlap between themselves, indicating that these four modules defined a single, larger cilia group consisting of 221 nuclear genes (Figure 6F). The genes making up these modules were highly co-expressed, with a fraction of genes identified in CiliaCut and the cilium proteome (Figure 6F). The intersection of the initial gene lists (modules, CiliaCut, overlap, and cilium proteome) defined a set of 44 genes, nine of which (*ODA1*, *DRC3*, *IFT121*, *IFT46*, *IFT74*, *MBO2*, *MIA1*, *PF16*, and *PF20*) were previously identified through forward genetic screens. We also extracted the co-expression cohorts associated with cilia modules, CiliaCut, and the cilium proteome (Figure 6G–I and Supplemental Data Sets S5, S11), linking several hundred genes to cilia. Their overlap (when using the N1 network) consisted of a set of 193 high-confidence cilia-related genes.

#### Photosynthesis modules

Four modules defined a larger photosynthesis group (Figure 6K) that we subdivided into three modules containing many of the genes encoding tetrapyrrole biosynthetic enzymes, while the last module was related to photosystems components. We extracted their co-expression cohorts (Figure 6L–N), resulting in hundreds of genes exhibiting strong co-expression. We also determined the overlap between the initial gene lists (Figure 6O) and their N1 cohorts (Figure 6P): the co-expression modules clearly included both photosynthesis- and tetrapyrrole-biosynthesis-related genes. As might be expected for genes necessary for proper chloroplast function, the overlap between N1 cohorts was substantial across all categories tested (modules, photosynthesis, and tetrapyrroles), highlighting interesting genes for potential follow-up studies within the modules and the N1 cohort (Supplemental Data Set S12).

### Genes in co-expression modules cluster based on their diurnal phase

**Figure 7:**
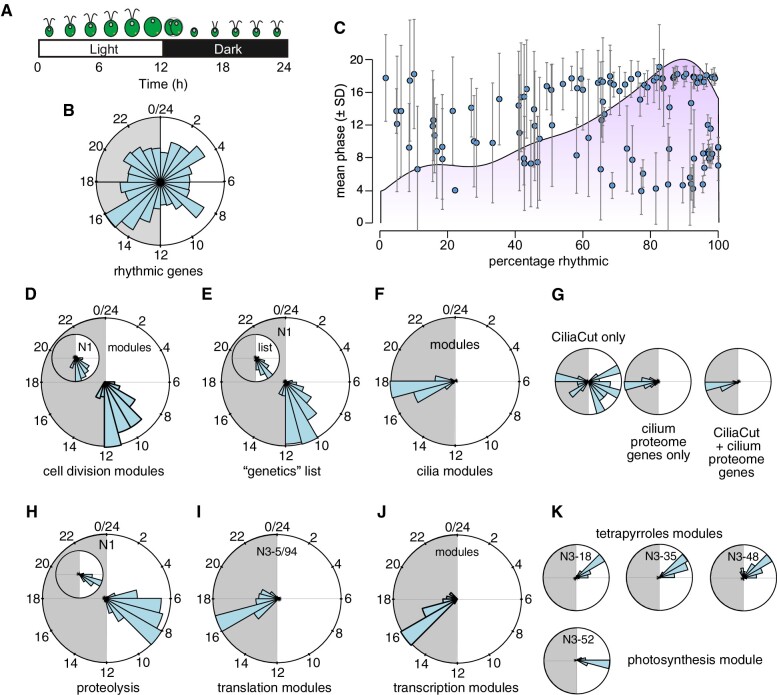
Co-expression modules routinely comprise genes with similar diurnal phases. **A)**, Schematic of the Chlamydomonas diurnal cycle in cell division events. **B)**, Phase distribution of 10,294 high-confidence diurnally rhythmic genes, shown as a circular plot covering the full 24 h of a complete diurnal cycle. Gray shade indicates night. **C)**, Co-expression modules with a high percentage of rhythmic genes exhibit a uniform diurnal phase. The light purple shade indicates the distribution of rhythmic modules. **D–K)**, Example of phase distribution for co-expression modules and associated N1 co-expression cohorts.

During our analysis of co-expression modules, we noticed a high proportion of diurnal synchronization between co-expressed genes within modules and their associated co-expression cohorts, even though diurnally expressed genes occupy the entire diurnal time landscape (Figure 7A and B). We therefore asked how frequently genes within co-expressed modules shared the same phase. Out of 117 modules extracted from the N3 network, 110 contained at least two rhythmic genes (Figure 7C), with a mean percentage of rhythmic genes of 65% and a median value of 71.6% (Figure 7C). Modules with few rhythmic genes tended to be associated with large standard deviations, indicative of little synchronization between the genes comprising them (Figure 7C). By contrast, modules consisting of a higher frequency of rhythmic genes showed high synchrony; their mean phase provided information relating to the biological function of each module, as illustrated below. Notably, the anti-correlated cohorts to most modules exhibited a mean phase that was 6–12 h out of phase with that of their related module (not shown), highlighting the importance of time-of-day when considering co-expression.

Molecular events leading to cell division are coordinately expressed with a phase distribution between 10 and 12 h after dawn: accordingly, we determined that the phase distribution of cell division modules and genes from the cell division “genetics” list showed the same phase preference (with 232 out of 245 genes being rhythmic) as did their associated co-expressed cohorts from the N1 network (Figure 7D and E). After cell division, cells reassemble cilia in anticipation of the coming dawn: 191 (out of 221) genes within cilia modules exhibited a marked preference for the middle of the night part of the diurnal cycle, which precisely corresponds to the time of cilia biogenesis (Figure 7F). The degree of synchrony may provide an additional selection criterion for co-expressed genes, as seen with phase distributions of genes belonging to CiliaCut only (i.e. CiliaCut genes whose gene products were not detected in the cilium proteome). Indeed, CiliaCut only genes displayed a wide range of diurnal phases, whereas co-expressed cilium proteome genes and genes at the intersection of CiliaCut and the cilium proteome were highly rhythmic and synchronized to the middle of the night (Figure 7).

We used the 96 genes (Figure 7H, inset) that form the protein degradation modules as baits to identify their co-expressed cohorts. They displayed a high degree of synchronized rhythmicity across diurnal datasets (Figure 7H). Only 2 out of the 96 genes from the protein degradation modules did not show rhythmic expression over a diurnal cycle. The occurrence of diurnal rhythmicity remained high in their associated co-expression cohorts, with 391 rhythmic genes out of 450. The distribution of their diurnal phases was also quite narrow for both sets of genes, with a peak in the second half of the day (Figure 7H). We speculate that timed protein degradation offers a mechanism for the removal of photo-oxidized proteins, which is broadly consistent with the recent characterization of Chlamydomonas mutants lacking activities for the E3 ubiquitin ligase and Cullin components of the SCF (Skip, Cullin, F-box) complex (Gabilly et al., 2019).

The majority of genes that belonged to the non-redundant translation modules N3-5/94 was rhythmic (121 out of 158), with diurnal phases concentrated in a narrow window of time between 3 and 5 h into the dark part of the diurnal cycle (Figure 7I). GO enrichment analysis indicated a role for these two modules in the nucleolus and ribosome biogenesis (Supplemental Table S3). Cytosolic *RPG*s were constitutively expressed and thus had no clear diurnal phase, whereas both plastid and mitochondrial *RPG*s exhibited preferred diurnal phases between 1–2 h and 3–5 h after dawn, respectively (Figure 7J), as expected (Zones et al., 2015).

Four modules defined a larger photosynthesis group that we subdivided into three modules containing many of the genes encoding tetrapyrrole biosynthetic enzymes, while the last module was related to photosystems components. Both sub-groups were highly rhythmic over the diurnal cycle and restricted to a small time window. Their respective phases agreed with their underlying biological function: genes encoding tetrapyrrole biosynthetic enzymes peaked ∼2 h prior to components of both photosystems (Figure 6K). While highly co-expressed, photosynthesis-, and tetrapyrroles-related modules did not substantially overlap (Supplemental Data Sets S4, S12), indicating that a diurnal phase difference of 2 h was sufficient to form independent clusters.

We conclude that co-expression modules are strongly influenced by the diurnal phase of their constituent genes. While this result may in itself not be surprising, it also raised the question of the overlap contribution of diurnal phase to clustering in our dataset, which we addressed next.

### Genes cluster based on their diurnal phase

While the majority of Chlamydomonas genes exhibits a diurnal expression profile when cells are grown under light–dark cycles, most of the samples included in our RNA-seq dataset were collected from cells grown in constant light, with the assumption that cells in such cultures would be largely asynchronous. Since we observed frequent co-expression that followed diurnal phase information, we determined whether genes globally clustered according to their diurnal phase, and whether cells in constant light retained some entrained properties.

**Figure 8:**
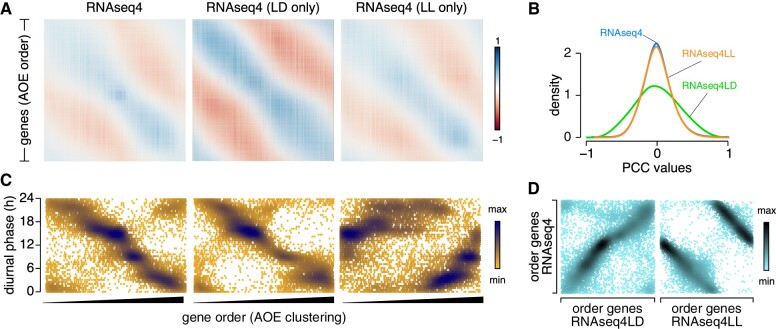
Genes cluster based on their diurnal phase. **A)**, Correlation matrix of the 17,741 Chlamydomonas nuclear genes, ordered based on clustering by the AOE method built into *corrplot*, using the fully normalized dataset RNAseq4, RNAseq4LD (consisting of RNA samples collected from cells grown under light-dark cycles), and RNAseq4LL (with all other RNA-seq samples) as input. **B)**, Distribution of pairwise PCCs for all gene pairs using RNAseq4, RNAseq4LD, and RNAseq4LL as input. **C)**, Scatterplot of diurnal phases from 10,294 high-confidence diurnally rhythmic genes, as a function of their order from AOE clustering, using RNAseq4, RNAseq4LD, and RNAseq4LL as input. We saved gene order following AOE clustering (from 1 to 17,741) and plotted the diurnal phase of the subset of 10,294 rhythmic genes (along the *y*-axis). D), Scatterplot of diurnal phases from 10,294 high-confidence diurnally rhythmic genes, ordered based on the AOE clustering method on RNAseq4 (*y*-axis) and RNAseq4LD or RNAseq4LL (*x*-axis).

We first explored how various clustering methods ordered genes as a function of their diurnal phase. We performed this analysis on three datasets: the fully normalized and complete dataset (RNAseq4), which included samples collected from cells grown in constant light and under diurnal cycles; RNAseq4LL, only consisting of samples collected from cells grown in constant light; and RNAseq4LD, comprising all samples with a rhythmic component, either diurnal or related to cell cycle progression. We calculated all pairwise PCCs and ordered genes according to hierarchical clustering (hclust, as shown in Supplemental Figure S5B), Angle of the Eigenvectors (AOE, Figure 8A), or FPC (Supplemental Figure S11). The AOE correlation matrix exhibited a smooth transition from the first gene to the last gene (along each row), with strong positive correlations along the diagonal and at the upper right corner, separated by a gradual transition to negative correlations parallel to the diagonal (Figure 8A). The matrix also lacked the localized clustering seen with the hclust method (compare Figure 8A with Supplemental Figure S5B). The FPC correlation matrix arranged pairwise PCCs in a similarly smooth pattern, with the strongest positive PCC values located in the upper left corner and the strongest negative PCCs in the upper right corner (Supplemental Figure S11A). The PCCs generated from RNAseq4LD followed a wider normal distribution relative to those of RNAseq4 and RNAseq4LL (Figure 8B), which we hypothesize results from the smaller number of samples and a higher amplitude in gene expression under rhythmic conditions, in contrast to averaged values from asynchronous cells.

We next assigned a row number to each gene according to their place within the AOE correlation matrices, from 1 to 17,741. For those that also exhibited a diurnal expression pattern (Supplemental Figure S9; Zones et al., 2015; Strenkert et al., 2019), we plotted their diurnal phase (on the *y*-axis) as a function of AOE gene order (on the *x*-axis). As shown in Figure 8C, the relationship between AOE gene order and diurnal phases was far from random, and instead followed a linear pattern, whereby genes that appeared first in the AOE correlation matrix had phases with peaks in the late evening. As gene row number increased, diurnal phases gradually decreased, demonstrating the widespread influence of diurnal phase on correlation potential between gene pairs. In addition, the overall pattern of the AOE correlation matrix was reminiscent of that seen for diurnal experiments (Figure 1C and E), with genes separated by 12 h in terms of diurnal phases showing the strongest anti-correlations, while genes in similar time neighborhoods shared strong co-expression.

The RNAseq4 and RNAseq4LD datasets globally resulted in the same gene order after AOE clustering (Figure 8C), which at first might imply that samples collected from diurnally grown cells imposed the observed gene ordering. However, this did not appear to be the case, as 1) the overall pattern of the AOE matrix for RNAseq4LL-derived PCC values was identical to that of RNAseq4 (Figure 8A), and 2) the corresponding gene order still carried diurnal information, as evidenced by the increase in diurnal phase with increasing gene order (Figure 8C), and despite the removal of all diurnal samples. Although the AOE clustering gene order did change between the RNAseq4 and RNAseq4LL matrices, the alteration in the pattern was systematic: a scatterplot of gene order for RNAseq4 and RNAseq4LL underscored the linear relationship between the two gene order series (Figure 8D). FPC clustering also sorted genes according to their diurnal phase, although along distinct parameters (Supplemental Figure S11B).

We conclude that diurnal phase contributes substantially to the clustering of genes, even for samples obtained from cells grown in constant light. Such samples appear to retain diurnal information that shapes the clustering outcome at the genome level.

### Molecular timetable analysis confirms residual synchronization of the Chlamydomonas transcriptome

That genes clearly clustered according to their diurnal phases even in a dataset comprised solely of samples collected from cells grown in constant light raised the possibility that these samples exhibit residual rhythmicity. We thus applied the molecular timetable method (Ueda et al., 2004) to all RNA-seq samples to determine the extent of rhythmicity they might show. The molecular timetable method, whose principle is briefly explained in Supplemental Figure S12, extracts the rhythmic (diurnal or circadian) information from single time-point transcriptomes using the known phases and expected expression levels from a reference diurnal (or circadian) dataset. We selected 480 genes across 24 phase bins; their peak time of expression is known exactly, as well as their expression levels. We then extracted their normalized expression from RNAseq4 and calculated the mean expression for each phase bin. Finally, we plotted this mean for each RNA-seq sample and each diurnal phase bin as a heatmap.

**Figure 9:**
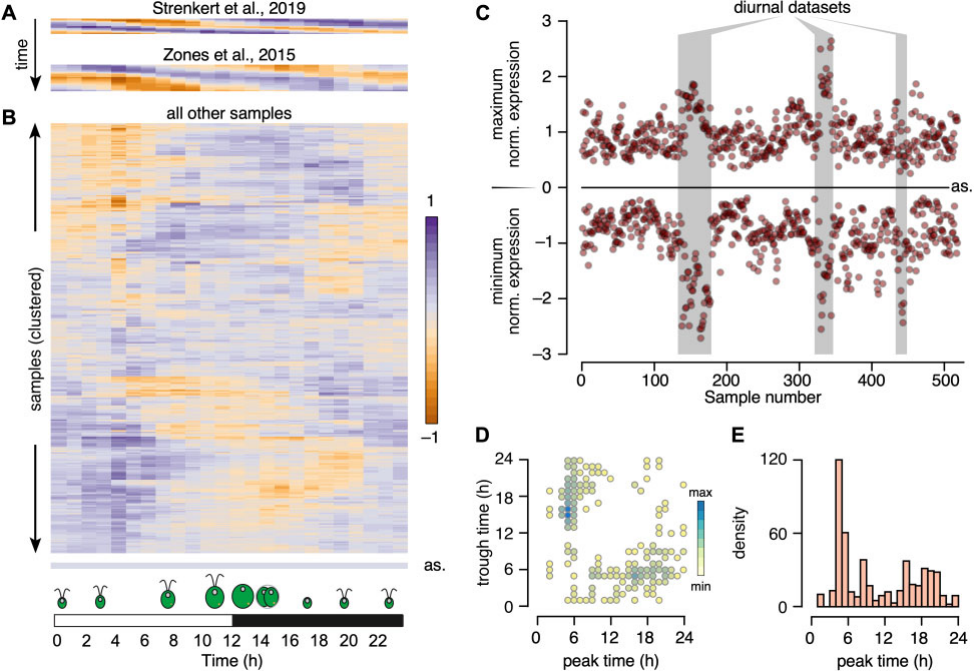
Chlamydomonas cultures grown in constant light retain substantial rhythmicity. **A)**, Heatmap representation of the molecular timetable approach, applied to two diurnal datasets: Strenkert et al. (2019) and Zones et al. (2015). **B)**, Heatmap representation of the molecular timetable approach, applied to all remaining RNA-seq samples. In panels (**A**) and (**B**), each sample is represented as the mean expression of 20 phase marker genes (per h). In (**A**), diurnal samples are ordered from top to bottom. For (**B**), samples were subjected to hierarchical clustering while generating the heatmap in R. as: heatmap from an asynchronous sample, corresponding to the average expression of all rhythmic genes for each time point. **C)**, Scatterplot of minimum and maximum normalized expression across all RNA-seq samples. Diurnal time courses are indicated by a gray shade. as: expected position of minima and maxima for a completely asynchronous sample. The samples are ordered by experiments, therefore consecutive data points belong to the same experiment. **D)**, Peak and trough times largely occur 12 h apart. Scatterplot of all peak expression time (*x*-axis) and trough times (*y*-axis). **E)**, Distribution of peak times across all RNA-seq samples.

We first looked at the two large diurnal time courses, shown in Figure 9A, to validate out methodology. Indeed, each diurnal sample (one row) showed a rhythmic pattern with each peak and trough separated by ∼12 h. In addition, successive time points were more similar to one another than to later time points, as observed earlier in the correlation matrix (Figure 1E). These results demonstrated the applicability of the molecular timetable method to Chlamydomonas RNA-seq samples, paving the way for the extraction of the internal time of the collected sample, as determined by the phase bin with maximal normalized expression.

We next subjected all remaining RNA-seq samples to the same analysis and clustered them based on their underlying pattern while generating the heatmap shown in Figure 9B. Completely asynchronous samples should appear off-white across all phase bins (“as,” bottom of Figure 9B); overwhelmingly, Chlamydomonas RNA-seq samples instead displayed remarkable residual rhythmicity. Diurnal time courses were easy to distinguish from other samples when we plotted the minimum and maximum normalized expression values associated with each sample (Figure 9C). Notably, most other samples, collected from cells grown in constant light, retained strong global oscillations, which we estimated to represent a synchronization between cells ranging from 21% to 96%, with a mean rhythmicity of 48%, based on the amplitude between minima and maxima relative to diurnal time course samples (Figure 9C).

The timing of minimum and maximum gene expression should be ∼12 h apart in diurnal and rhythmic samples: we therefore plotted peak and trough times predicted for all samples based on the molecular timetable data. As shown in Figure 9D, most samples indeed reached peak value 12 h after their lowest time-point, validating our hypothesis that the majority of Chlamydomonas RNA-seq samples exhibit strong residual rhythmicity even when the cells were grown in constant light.

Finally, we asked whether samples displayed a preferential diurnal phase by plotting the distribution of peak phases across all samples. To our surprise, about one third of all samples showed a peak phase between 5 and 6 h after dawn.

### Applicability of the molecular timetable method to other algae: *V. carteri* and *C. zofingiensis* as tests

**Figure 10:**
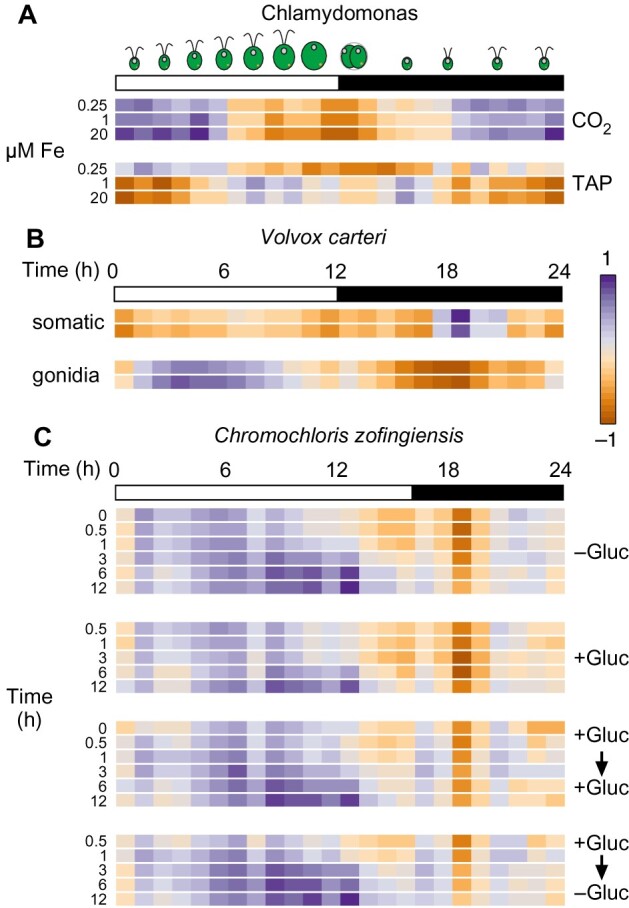
Application of the molecular timetable method to independent RNA-seq experiments across algae. **A)**, Reanalysis of a transcriptome dataset included in our initial RNA-seq data (Urzica et a., 2012b). We subjected FPKM values to log_2_ normalization, followed by normalization to the mean (obtained during the normalization steps that yielded RNAseq4). We then used the molecular timetable method to determine the rhythmic pattern of the samples (Chlamydomonas CC-4532 strain grown in Tris Acetate Phosphate (TAP) or Tris Phosphate (CO_2_) medium with 0.25, 1, or 20 µM FeEDTA). **B)**, Molecular timetable method applied to *V. carteri* samples collected in duplicates from somatic or gonidial cells (Matt and Umen, 2018). **C)**, Molecular timetable method applied to *C. zofingiensis* samples collected over 12 h after addition and removal of glucose (Roth et al., 2019). For (**A**), we used 960 highly rhythmic genes to draw the heatmap. For (**B**) and (**C**), we included all rhythmic genes with orthologs in *V. cateri* (**B**) or *C. zofingiensis* (**C**), after log_2_ normalization and normalization with the Chlamydomonas-derived gene means.

Incorporating new Chlamydomonas transcriptome datasets to the one we used here would be cumbersome, as it would entail repeating all normalization steps each time a new dataset is added. A more practical approach would be to subject new transcriptome datasets to an abridged normalization, namely log_2_ normalization followed by normalization to the mean calculated from our full dataset. We tested the usefulness of this method by reanalyzing a transcriptome dataset included in our original list that was focused on iron homeostasis (Urzica et al., 2012b), for which Chlamydomonas cells had been grown with various iron concentrations (0.25, 1, or 20 µM FeEDTA) in autotrophic (no reduced carbon source provided, but cultures were bubbled with CO_2_) or heterotrophic (with acetate as reduced carbon source) conditions. We normalized FPKM counts to the mean inferred from the full RNA-seq dataset, and used the respective diurnal phase for each gene (Supplemental Data Set S13). As shown in Figure 10A, autotrophic cultures exhibited a similar molecular timetable profile, with an estimated internal phase around dawn across all three iron concentrations. In sharp contrast, heterotrophic cultures responded very differently: indeed, iron-limited cultures (0.25 µM FeEDTA) were 12 h out of phase with the other two samples. Iron-limited heterotrophic cultures grow more slowly than iron-deficient (1 µM FeEDTA) or iron-replete cultures (20 µM FeEDTA). We hypothesize that the difference in internal phase between heterotrophic samples may thus partially reflect the time at which cultures were sampled, as cells were harvested at the same cell density (Urzica et al., 2012b). However, we cannot exclude a contribution to a slower circadian clock under low iron conditions, as described for land plants (Chen et al., 2013; Hong et al., 2013; Salomé et al., 2013). Nonetheless, we conclude that the molecular timetable method is applicable to Chlamydomonas samples after performing log_2_ and mean normalization.

We then explored the applicability of this method to other algae where a high-density diurnal time course is not available: *Vovox carteri* and *C. zofingiensis*. The molecular timetable method requires two sets of information: the mean expression and standard deviation of a given gene for normalization; the predicted diurnal or circadian phase of the gene. However, both algal strains (*V. carteri* and *C. zofingiensis*) presently lack a high-density expression estimate across a diurnal time course. Therefore, we transferred the mean, standard deviation, and known diurnal phase of all Chlamydomonas genes over to their putative one-to-one orthologs, as determined in BioMart at Phytozome.

*Vovox carteri* samples consisted of two technical replicates each collected from somatic and gonidial cells (Matt and Umen, 2018). We obtained one-to-one orthologs between Chlamydomonas and *V. carteri* from Phytozome, after which we subjected all *C. carteri* genes with a rhythmic Chlamydomonas ortholog to log_2_ normalization and to normalization with Chlamydomonas means. We then calculated the average normalized expression for all genes, in 1-h bins. Gonidial cells appeared strongly rhythmic, with a peak phase around 4–5 h after dawn and a trough ∼12 h later (Figure 10B). Remarkably, somatic cells exhibited a completely different profile with a peak phase in the middle of the night. We performed the same analysis of transcriptome samples collected in *C. zofingiensis* over a 12-h time course with addition or removal of glucose from the growth medium (Roth et al., 2019). Here, cultures were maintained in light-dark cycles consisting 16 h light and 8 h darkness. All samples exhibited a rhythmic profile, strongly indicating that the molecular timetable accurately predicted the internal phase of the samples. Indeed, the peak phase of samples collected later during the day showed a clear and distinct shift to a later phase. Notably, the rhythmic pattern extracted from these transcriptome samples followed the same overall pattern regardless of the treatment imposed on the cultures, which is consistent with the strong contribution of time-of-day noted in these samples (Roth et al., 2019).

We conclude that the molecular timetable method can be applied to Chlamydomonas and to other algae, even when they lack a reference diurnal time course. Such analysis would allow a rapid estimation of the contribution of rhythmic gene expression to variation in gene expression, even in the absence of a reference diurnal time course.

## Discussion

The assembly of 518 RNA-seq samples into one data set offers a unique opportunity to explore the transcriptome landscape in Chlamydomonas. We exploited this data set to determine whether independent experiments exhibit the same transcriptome profile (they largely do not), whether genes follow similar expression trajectories (they sometimes do), and what factors might contribute to their co-clustering (diurnal time plays a significant role). The analyses presented here likely only skim the surface of extractable information; we invite others to use this dataset for their own research questions.

We were surprised to see how little correlation existed between Chlamydomonas experiments, even though several queried the same biological question, such as responses to nitrogen deficiency or metal deficiencies (Figure 2). Samples collected in the same laboratory similarly failed to show strong correlations, although growth conditions are likely to be similar. We do not fully understand the underlying source of variation, but we propose that strong residual rhythmic gene expression may contribute to the observed pattern. As a test of our analysis pipeline, we determined the correlation matrix of Arabidopsis microarray data sets, downloaded from AtGenExpress. As shown in Supplemental Figure S13, samples (using the expression data for all genes as data points) clearly grouped as a function of the tissue of origin, with shoot and leaf samples generally strongly correlated, while anti-correlated with root samples. It is likely that Arabidopsis samples show strong differentiation of their expression profiles as a function of the tissue of origin, as might be expected, thus validating our pipeline.

Co-expression modules assemble the most consistent gene pairs into a coherent list that is characterized by high connectivity between genes (Supplemental Figure S8B). However, each gene is itself co-expressed with many genes that do not necessarily meet the interconnectivity requirements for assignment to a module (Supplemental Figure S8A), here referred to as co-expression cohorts. The co-expression cohorts can nevertheless provide clues as to the function of a gene, especially when it does not belong to a module. In addition, genes with the opposite expression profile can give hints as to the function of a gene of interest. We have extracted co-expression and anti-correlation cohorts for all Chlamydomonas genes, provided as Supplemental Data Sets S4–S9. We also provide the scripts used here as Supplemental Protocols. We hope that this type of analysis spurs new discoveries, not only in Chlamydomonas but also in Arabidopsis and other plants. Our results with Arabidopsis *RPG*s (Figure 4E) demonstrate the applicability of the method to other organisms.

We expect that the resource presented here will be combined with the output from other high-throughput approaches (Li et al., 2015, 2019; Vilarrasa-Blasi et al., 2020) to ascertain gene function and/or prioritize genes for further functional studies.

The Chlamydomonas life cycle resolves around cell division, the timing of which can be synchronized to dusk by light–dark cycles (Cross and Umen, 2015; Zones et al., 2015; Strenkert et al., 2019). When maintained under entraining conditions, at least 80% of the Chlamydomonas transcriptome exhibits rhythmic expression. It is unclear how quickly algal cells become asynchronous when transferred to constant light conditions. It is thought that cultures grown in constant light are largely arrhythmic at the population level due to loss of synchrony. When applying the molecular timetable to Chlamydomonas RNA-seq samples, we discovered that the majority of samples exhibited substantial rhythmicity, even when collected from cultures grown in constant light (Figure 9). About one third of all samples appeared to have been collected 5–6 h after subjective dawn (i.e. the dark-to-light transition had the cells been maintained under entraining conditions). Based on the amplitude between minima and maxima extracted from phase marker genes, we estimate that 21–96% of cells within a given culture were synchronized, with a mean of 48%. Chlamydomonas strain stocks are typically kept in constant light on solid medium before inoculating a liquid culture, which will itself be placed in constant light. Pre-cultures are common before inoculating the test culture; cells are generally collected by centrifugation when they reach mid-log. It is therefore possible that diluting cells at the beginning of an experiment sends a resetting signal to Chlamydomonas diurnal rhythms, the signature of which is still present 2–3 days later, as evidenced by the degree of residual synchronization in all samples analyzed. Another possible explanation would call upon social signaling (or quorum sensing) between Chlamydomonas cells (Asfahl and Schuster, 2017). In such a mechanism, cells may secrete signaling molecules or pheromones that inform other cells of their metabolic state. Alternatively, cells may secrete and share metabolic intermediates, which could also accomplish synchrony. Chlamydomonas cultures can secrete agonists of bacterial quorum sensing (Teplitski et al., 2004), but whether such compounds have any effect on synchronization of algal cultures has not been investigated.

We are only seeing the bulk behavior of Chlamydomonas cultures in this data set. Only a single-cell RNA-seq (scRNA-seq) analysis will allow a more detailed dissection of the diurnal contribution to the Chlamydomonas transcriptome landscape. To begin to explore this possibility, we recently performed scRNA-seq on almost 60,000 Chlamydomonas cells grown under three conditions and from two genotypes. Indeed, we observed a substantial heterogeneity among the cells that was partially explained by the endogenous phase of individual cells (Ma et al., 2021). Although cultures were grown in constant light for several weeks, we hypothesize that diluting cells at the beginning of an experiment may act as a resetting signal for the endogenous cell cycle and other daily rhythms.

Our observations also raise a question regarding the design of RNA-seq experiments when assessing the effect of a mutation or a treatment on cultures: Is it more important to collect samples at the same cell density or at the same time? Our results suggest that sampling time exerts a far greater influence on expression outcomes than sampling density would. Best practices for RNA-seq analysis may therefore dictate that a matched control sample be collected at each time-point in order to remove any contribution to differential gene expression from the strong rhythmicity exhibited by cultures. Genes belonging to the same co-expressed modules tended to have the same diurnal phase (Figure 9C); the narrow window of expression seen in rhythmic genes would thus be missed when comparing samples collected hours apart. In Arabidopsis, samples collected 30 min apart already exhibited differential expression (Hsu and Harmer, 2012). Our results generalize this observation.

The molecular timetable method is a powerful and easily implemented method to test the rhythmic component of transcriptome data. We demonstrate here that Chlamydomonas data can be transferred onto other algae like *V. carteri* and *C. zofingiensis* to reveal an unexpected dimension of rhythmic expression from single time points. We propose that all transcriptome datasets should be subjected to such analysis before delving into more in depth analysis, to estimate the fraction of variation in gene expression that might be explained by rhythmic expression. We provide the mean and phase values from Chlamydomonas to normalize RNA-seq data from other algae as Supplemental Data Set S13.

In conclusion, we describe here an analysis of co-expression in the green unicellular alga Chlamydomonas. We observed known and new connections between genes ad provide the tools to take this analysis further for any gene of interest, in both Chlamydomonas and other system with a body of transcriptome data available.

## Materials and methods

### Co-expression analysis network in Chlamydomonas

We reanalyzed a set of 58 RNA-seq experiments, consisting of 518 samples, by mapping reads to version v5.5 of the Chlamydomonas genome (v5.5 from Phytozome) with STAR (v2.5) (Dobin et al., 2013) using default settings except—alignIntronMax 10000—outFilterMismatchNoverLmax 0.04. Expression was calculated in terms of fragments per kb per million mapped reads (FPKMs) with cuffdiff (v2.0.2) (Trapnell et al., 2014) using default settings except—multi-read-correct—max-bundle-frags 1000000000. We assembled all expression estimates as FPKM into one file and did not attempt to correct for batch effect at this stage, with the thought that such effects would contribute to the variation in expression. We then normalized the resulting expression data set (Supplemental Protocol S1). First, we log_2_-transformed mean FPKMs across replicates was with a pseudo-count of “1” added prior to conversion, followed by quantile normalization with the R package *preprocessCore*. Finally, we subtracted mean expression across all experiments for each gene, which removed any potential batch effects from the data. We calculated PCCs with the *cor()* function in R and visualized them for each gene pair using the R package *corrplot*, using all 518 expression estimates. We maintained four expression datasets following each normalization step: RNAseq1 (mean FPKMs); RNAseq2 (log_2_-normalized); RNAseq3 (quantile-normalized); and RNAseq4 (normalized to mean).

We calculated the rank for all gene pairs (Supplemental Protocol S2) by inverting the sign of PCCs by multiplying the data frame by –1, then converting PCC values for each gene into ranks with the function *rank()* in R. We derived the MRs for two genes a and b from the formula MR(a,b) = √(rank_a→b_ × rank_b→a_). Considering a matrix of ranks, the ranks rank_a→b_ and rank_b→a_ are geometrically linked on either side of the diagonal: if rank_a→b_ has the coordinates (*x*,*y*) in the rank matrix, then rank_b→a_ will have the coordinates (*y*,*x*). We therefore transposed the rank matrix with the *t()* function in R. We obtained MR values for each gene pair by multiplying each cell from the rank matrix by their counterpart in the transposed rank matrix, then square-rooted.

For network selection and visualization, we calculated edge weights from MR values with the formula: *Nx* = e^−(MR−1)/^^*x*^, with *x* = 5, 10, 25, 50, or 100 (Supplemental Protocol S2 for networks 1–3). Only *Nx* ≥ 0.01 were considered significant. We extracted gene pairs with significant edge weights from the full edge weight matrix and loaded them into Cytoscape 3.5.1. We detected modules of co-expressed genes with ClusterONE with default parameters and saved the modules as a .csv file, which includes the *P*-value associated with each module. Modules with a *P*-value ≤ 0.1 were considered significant.

We also determined lists of anti-correlated genes by ranking PCC values from the non-inverted PCC matrix generated by *corrplot*, and by calculating associated edge weights as above (Supplemental Protocol S3). In this case, we limited our analysis to identifying anti-correlated genes, as ClusterONE cannot detect modules using edge weights from anti-correlated genes.

### Co-expression analysis network in Arabidopsis

Microarray datasets were downloaded from the AtGenExpress project site (http://jsp.weigelworld.org/AtGenExpress/resources/), and collated into a single file that consisted of 34 Arabidopsis accessions, 16 sets of etiolated seedlings exposed to various light treatments, 36 sets of seedlings exposed to pathogens, 13 cell culture samples, 68 sets each for shoots and roots exposed to various abiotic stresses, 79 developmental samples (72 from shoots or leaves, 7 from roots), and 18 sets each for leaves and roots subjected to iron deficiency, with controls included. We removed all control probes from the data set, bringing the number of probes on the arrays from 22,810 to 22,746. We log_2_-normalized all data when not already done, and followed the same normalization steps described for the Chlamydomonas data set.

### Analysis of co-expression from ClusterONE modules

We extracted normalized expression data (from RNAseq4) for genes belonging to a given cluster in R using the *stack()* and *unstack()* functions, and generated the corresponding co-expression matrix with *corrplot* (Supplemental Protocol S4). We tested for overlap between co-expression modules with similar predicted function with the online tool Venny (Oliveros, 2007), and redrew co-expression matrices with a non-redundant gene list as input. Unless stated otherwise, we ordered genes based on the FPC clustering method built into *corrplot* (Supplemental Protocol S4).

### Analysis of co-expression from manually curated and community gene lists

We extracted normalized expression data for genes that belonged to manually curated or community-generated lists as described above for co-expression modules (Supplemental Protocol S4). We maintained the same gene order when working with community lists, as the genes were sorted and grouped based on shared function. We sorted genes from manually curated lists following the FPC method in *corrplot*.

For *RPG*s from Arabidopsis, we downloaded a list of 429 *RPG*s identified in the Arabidopsis genome (Sormani et al., 2011). Of those, 357 were represented by a probe on the ATH1 Affymetrix microarray and were predicted to encode ribosomal proteins localizing to the cytosol (184), mitochondria (55), chloroplasts (69), or with unclear localization (49, including 13 with a predicted nuclear location). We extracted the normalized expression data for all genes and performed hierarchical clustering (hclust method in *corrplot*) on each *RPG* subgroup. We then reordered all *RPG*s represented on the Affymetrix arrays according to FPC clustering order and recalculated the correlation matrix.

### Analysis of histone gene expression and genome organization across a subset of the green lineage

Most histone transcripts are not polyadenylated; we therefore split our data set into RNA-seq experiments that were subjected to ribodepletion or with histone expression >5 FPKMs (4 experiments, or 36 samples, including the diurnal time course from (Strenkert et al., 2019)) and all remaining 480 samples. We then normalized the two data sets separately as described in Supplemental Protocol S1 and Supplemental Figure S1 and plotted their respective correlation matrices, while maintaining histone genes ordered based on their chromosomal positions.

We noticed that the order of histone genes was not random in the Chlamydomonas genome. We determined the orientation of all gene pairs by visual inspection in GBrowse at Phytozome. To identify histone genes in Arabidopsis, *M. polymorpha*, and *Physcomitrium patens*, we performed BLASTP searches at NCBI or Phytozome using the protein sequence for one Histone H2A, H2B, H3, and H4 as query, followed by ordering of all histone genes based on their unique locus identifier. To identify histone genes in *O. lucimarinus*, *C. zofingiensis*, *D. salina*, *Micromonas* sp., and *V. carteri*, we followed the same steps described above but with a Chlamydomonas histone protein as query. The current version of the *D. salina* genome lacks annotated histone H2B, prompting us to perform a TBLASTN search against the *D. salina* genome (translated in all six open reading frames) with Chlamydomonas Histone H2B as query, thus identifying 19 putative *Histone H2B* loci. Since they have no locus identifier, we looked for the closest gene model in GBrowse at Phytozome, revealing 13 *Histone H2A* genes in a divergent orientation with the *Histone H2B* loci.

### Identification of co-expression cohorts

We extracted the sets of genes co-expressed with each gene belonging to any gene list in R by merging each query gene list with a file representing all nodes and edges from networks N1 to N3 (Supplemental Protocol S5). We collapsed each co-expression cohort into a non-redundant list by using the function *unique()* in R, since genes that share the same expression profile will be part of each other’s co-expression cohort. We then tested each subset from networks N1 to N3 for overlap with *merge()* or *join()* in R and Venny (Oliveros, 2007).

Manually curated and community-generated gene lists presented an initial challenge, since not all of their constituents are necessarily co-expressed (e.g. only a fraction of the genes defined by the mutant screen carried out by Dr. Frederic Cross for cell cycle mutants is co-expressed). We therefore 1) ordered genes using the FPC clustering method; 2) counted how many gene pair PCCs were above 0.25, 0.4, or 0.5 for each row of the matrix in order to 3) define cut-offs between subsets of genes with high, medium, or low PCCs. We then used these subsets (from 1 to 3) as bait to identify their associated co-expression cohort (Supplemental Protocol S6).

### GO category enrichment in co-expressed modules

We tested our co-expression modules for Gene Ontology term enrichment by using the PANTHER database (pantherdb.org) through the Gene Ontology Resource page (http://geneontology.org). First, all Chlamydomonas gene identifiers (Crexx.gxxxxxxx) were converted to their corresponding Uniprot identifiers using a gene-to-Uniprot list generated in-house. Of 117 modules, 86 retained at least 10 genes with corresponding Uniprot identifiers (31 had ≤9 genes with matching Uniprot identifiers and were deemed too small for further analysis), and 37 returned significant enrichment in GO term(s) for Biological Process. We subjected each gene list (as Uniprot IDs) to GO term enrichment analysis by running the analysis on the PANTHER website manually.

### Venn diagrams and gene list overlaps

We compared gene lists and determined the extent of overlap with the online tool Venny (Oliveros, 2007). Proportional Venn diagrams were drawn with BioVenn (Hulsen et al., 2008) for two-way diagrams or EulerAPE 3.0.0 (Micallef and Rodgers, 2014) for three-way diagrams.

### Determination of diurnal phase distribution across gene lists

We first generated a list of high-confidence rhythmic genes over the diurnal cycle by selecting genes that were deemed rhythmic from two recent diurnal studies in Chlamydomonas (Zones et al., 2015; Strenkert et al., 2019). Since the two studies used different reference points as time zero, we corrected the diurnal phases from Zones by shifting them by 12 h, with manual editing for diurnal phase values that were larger than 24 h (a phase of 26 h is identical to a phase of 2 h, e.g.). We then selected rhythmic across both studies by using the *merge()* function in R, followed by *na.omit()* to remove any gene that was rhythmic in only one of the two lists. We then extracted the subset of genes with a diurnal phase with the *merge()* function in R. The resulting list of diurnal phases was then used as input for the circular R package to draw the distribution of phases in a circle plot (Supplemental Protocol S7).

For the plots of diurnal phase as a function of clustering order, we saved the order of genes following clustering of the entire gene matrix by the AOE or FPC clustering methods and turned it into a rank (from 1 to 17,741). We then matched each rank with the diurnal phase of the corresponding gene and generated the plots, using *densCols()* in R to avoid over-plotting.

### Molecular timetable method

For the analysis of Chlamydomonas data, we selected 960 highly rhythmic genes, consisting of 20 genes per 1/2 h phase bins calculated from JTK_CYCLE with the lowest BH.Q *P*-value (Strenkert et al., 2019) to act as phase markers along the diurnal cycle. According to the molecular timetable method (Ueda et al., 2004), normalized transcript levels for genes measured in truly asynchronous samples (in this case, cultures) will tend to hover around zero, with no obvious pattern. Transcript levels from samples with partial synchrony across cells will however exhibit a clearly identifiable pattern when their normalized expression is ordered as a function of their expected diurnal phase (see also Supplemental Figure S12). We therefore extracted the normalized expression data from the data set RNAseq4 for the 960 markers genes, after which we calculated the mean normalized expression of genes within the same phase bins, and visualized the results as a heatmap (Supplemental Protocol S8). We also determined the amplitude of the underlying diurnal pattern by defining the minima and maxima of mean normalized expression data across all phase bins.

For the analysis of RNA-seq data from *V. carteri* and *C. zofingiensis*, we first downloaded the list of orthologs between Chlamydomonas and *V. carteri* or *C. zofingiensis* from BioMart at Phytozome v12. We also downloaded transcript lengths for *V. carteri*, as the one RNA-seq data set available (GSE104835) reports raw counts rather than FPKMs. We then converted raw counts to FPKMs, retained only those genes in *V. carteri* with a one-to-one ortholog in Chlamydomonas (7,377 genes), removed genes whose Chlamydomonas ortholog was not rhythmic (1,840 genes), and calculated the mean normalized expression of *V. carteri* genes within each phase bin, as predicted by the diurnal phase of their Chlamydomonas orthologs, and plotted the results as a heatmap (Supplemental Protocol S9).

We used a previously published expression data set for *C. zofingiensis* (Supplemental Data Set S1 from Roth et al., 2019). The FPKM values were log_2_-normalized in Excel before saving the file as a .txt file for import into R. Separately, we determined the list of *C. zofingiensis* genes with a Chlamydomonas one-to-one ortholog (2,351 genes) and retained those with a rhythmic Chlamydomonas ortholog (1,541 genes). We then merged the two files, normalized *C. zofingiensis* expression values (log_2_[FPKM + 1]) using the mean expression of their Chlamydomonas orthologs, averaged in phase bins according to the diurnal phase of the Chlamydomonas orthologs, and plotted as a heatmap (Supplemental Protocol S10).

### Statistics

PCC values for the entire genome were calculated with the *cor* function in R, and their distributions plotted with the *density* function in R. A random normal distribution of mean = 0 and standard deviation = 0.2 was generated with the *rnorm* function in R for 100 million values; only 23 values fell outside of the –1 to +1 range and were not discarded.

For comparisons between distributions, we applied a Kolmogorov–Smirnov test (ks-test) using the *ks.test* function in R.

## Supplemental data

Please note: Supplemental Data Sets 1-13, Supplemental Files 1-9, and the Supplemental Protocols are available at datadryad.org with the DOI: https://doi.org/10.5068/D1WD55.

**Supplemental Figure S1.** Normalizations of the Chlamydomonas transcriptome dataset.

**Supplemental Figure S2.** How *RPGs* respond to each normalization step.

**Supplemental Figure S3.** The R package *corrplot* and visualization of large correlation matrices.

**Supplemental Figure S4.** Correlations between experimental samples and normalization methods.

**Supplemental Figure S5.** Chlamydomonas gene pairs are largely not co-expressed.

**Supplemental Figure S6.** Testing known patterns of co-expression in the RNAseq4 data set.

**Supplemental Figure S7.** From co-expression cohorts to co-expression modules.

**Supplemental Figure S8.** Using module nodes as baits to identify co-expressed genes.

**Supplemental Figure S9.** Convergence of diurnal phase between two time-courses.

**Supplemental Figure S10.** Co-expression of the protein degradation machinery is limited to the 26S proteasome.

**Supplemental Figure S11.** Genes cluster based on their diurnal phase.

**Supplemental Figure S12.** Molecular timetable method to extract diurnal information from single time-points.

**Supplemental Figure S13.** Arabidopsis microarray data clearly differentiate between tissue types.

**Supplemental Table S1.** Summary of expression estimates across all conditions and samples

**Supplemental Table S2.** Cohort and modules sizes for co-expression data derived from the RNAseq4 dataset

**Supplemental Table S3**. Summary of GO terms enriched in N3 co-expressed clusters

**Supplemental Data Set S1.** *RPGs* in Chlamydomonas, ordered by the final location of their products.

**Supplemental Data Set S2.** Genes used to test known patterns of co-expression.

**Supplemental Data Set S3.** Chlamydomonas respiratory complex genes.

**Supplemental Data Set S4.** Photosynthesis and tetrapyrrole biosynthesis genes.

**Supplemental Data Set S5.** Genes from CiliaCut and the cilium proteome.

**Supplemental Data Set S6.** Arabidopsis ribosome protein genes.

**Supplemental Data Set S7.** Histone genes in Chlamydomonas.

**Supplemental Data Set S8.** Histone genes in selected algae and plants.

**Supplemental Data Set S9.** Cell division modules and their co-expressed cohorts.

**Supplemental Data Set S10.** Protein degradation, proteasome, and their co-expressed cohorts.

**Supplemental Data Set S11.** Cilia genes, sorted by their overlap with CiliaCut, and their level of co-expression.

**Supplemental Data Set S12.** Photosynthesis modules and their co-expressed cohorts.

**Supplemental Data Set S13.** Mean and diurnal phase of Chlamydomonas genes for the timetable method.

**Supplemental File S1.** The fully normalized RNA-seq dataset.

**Supplemental File S2.** List of co-expressed genes for each nuclear Chlamydomonas gene for the N1 network.

**Supplemental File S3.** List of co-expressed genes for each nuclear Chlamydomonas gene for the N2 network.

**Supplemental File S4.** List of co-expressed genes for each nuclear Chlamydomonas gene for the N3 network.

**Supplemental File S5.** List of anti-correlated genes for each nuclear Chlamydomonas gene for the N1 network.

**Supplemental File S6.** List of anti-correlated genes for each nuclear Chlamydomonas gene for the N2 network.

**Supplemental File S7.** List of anti-correlated genes for each nuclear Chlamydomonas gene for the N3 network.

**Supplemental File S8.** The fully normalized Arabidopsis dataset.

**Supplemental File S9.** List of genes from the 117 co-expression modules identified in network N3.

**Supplemental Protocols.** Scripts to turn RNA-seq data sets into MRs, gene co-expression cohorts, and co-expression modules.

## Supplementary Material

koab042_Supplementary_Data

## Notes

P.A.S. designed and conducted all analyses with supervision from S.S.M. P.A.S. wrote the manuscript.

The author responsible for distribution of materials integral to the findings presented in this article in accordance with the policy described in the Instructions for Authors (https://academic.oup.com/plcell) is: Patrice A. Salomé (salome@chem.ucla.edu).
