# Supplementary material for: Co-expression networks in Chlamydomonas reveal significant rhythmicity in batch cultures and empower gene function discovery
Source: Plant Cell. 2021 Feb 2;33(4):1058–82. doi: 10.1093/plcell/koab042 (PMC8226298; doi:10.1093/plcell/koab042)
Supplement: koab042_Supplementary_Data [file koab042_supplementary_data.zip › tpc.00822.2020-s03.pdf]

Supplemental Data. Salomé and Merchant (2021). Co-Expression Networks in Chlamydomonas Reveal Significant Rhythmicity in Batch Cultures and Empower Gene Function Discovery. Plant Cell.

The following supplemental data files were submitted to the Data Dryad Repository and are available at https://doi.org/10.5068/D1WD55.

Supplemental Data Set 1. Ribosomal protein genes (*RPG*) in Chlamydomonas, ordered by the final location of their products.
Supplemental Data Set 2. Genes used to test known patterns of co-expression.
Supplemental Data Set 3. Chlamydomonas respiratory complex genes.
Supplemental Data Set 4. Photosynthesis and tetrapyrrole biosynthesis genes.
Supplemental Data Set 5. Genes from CiliaCut and the cilium proteome.
Supplemental Data Set 6. Arabidopsis ribosome protein genes.
Supplemental Data Set 7. Histone genes in Chlamydomonas.
Supplemental Data Set 8. Histone genes in selected algae and plants.
Supplemental Data Set 9. Cell division modules and their co-expressed cohorts.
Supplemental Data Set 10. Protein degradation, proteasome and their co-expressed cohorts.
Supplemental Data Set 11. Cilia genes, sorted by their overlap with CiliaCut and their level of co-expression.
Supplemental Data Set 12. Photosynthesis modules and their co-expressed cohorts.
Supplemental Data Set 13. Mean and diurnal phase of Chlamydomonas genes for the timetable method.
Supplemental File 1. The fully normalized RNA-seq dataset.
Supplemental File 2. List of co-expressed genes for each nuclear Chlamydomonas gene for the N1 network.
Supplemental File 3. List of co-expressed genes for each nuclear Chlamydomonas gene for the N2 network.
Supplemental File 4. List of co-expressed genes for each nuclear Chlamydomonas gene for the N3 network.
Supplemental File 5. List of anti-correlated genes for each nuclear Chlamydomonas gene for the N1 network.
Supplemental File 6. List of anti-correlated genes for each nuclear Chlamydomonas gene for the N2 network.
Supplemental File 7. List of anti-correlated genes for each nuclear Chlamydomonas gene for the N3 network.
Supplemental File 8. The fully normalized Arabidopsis dataset.
Supplemental File 9. List of genes from the 117 co-expression modules identified in network N3.
Supplemental Protocols. Scripts to turn RNA-seq data sets into mutual ranks, gene co-expression cohorts and co-expression modules.

<u>Supplemental Materials in this file:</u>

Supplemental Figure 1. Normalizations of the Chlamydomonas transcriptome dataset.
Supplemental Figure 2. How ribosomal protein genes (RPGs) respond to each normalization step.
Supplemental Figure 3. The R package corrplot and visualization of large correlation matrices.
Supplemental Figure 4. Correlations between experimental samples and normalization methods.
Supplemental Figure 5. Chlamydomonas gene pairs are largely not co-expressed.
Supplemental Figure 6. Testing known patterns of co-expression in the RNAseq4 data set.
Supplemental Figure 7. From co-expression cohorts to co-expression modules.
Supplemental Figure 8. Using module nodes as baits to identify co-expressed genes.
Supplemental Figure 9. Convergence of diurnal phase between two time-courses.
Supplemental Figure 10. Co-expression of the protein degradation machinery is limited to the 26S proteasome.
Supplemental Figure 11. Genes Cluster Based on their Diurnal Phase.
Supplemental Figure 12. Molecular timetable method to extract diurnal information from single time-points.
Supplemental Figure 13. Arabidopsis microarray data clearly differentiates between tissue types.
Supplemental Table 1. Summary of expression estimates across all conditions and samples.
Supplemental Table 2. Cohort and modules sizes for co-expression data derived from the RNAseq4 dataset.
Supplemental Table 3. Summary of GO terms enriched in N3 co-expressed clusters.

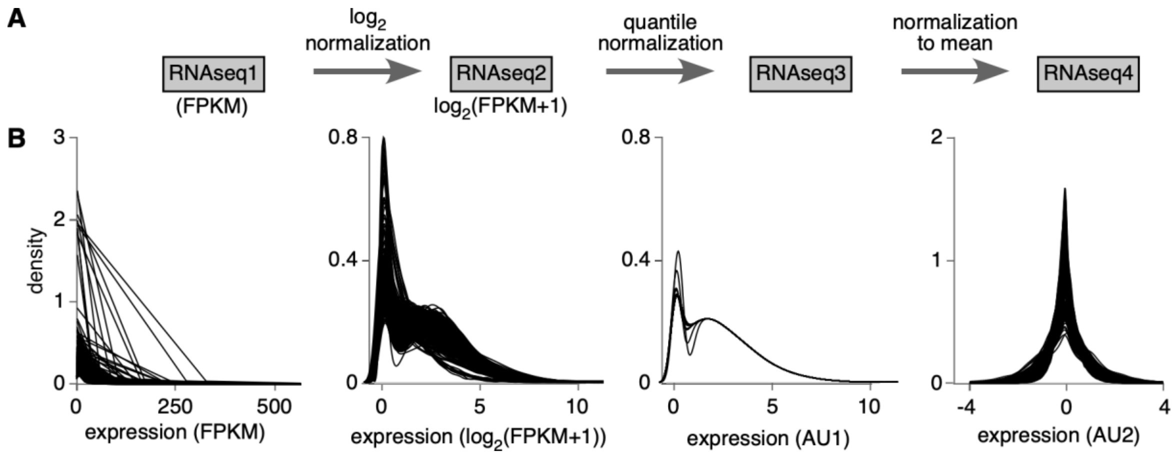

Supplemental Figure 1. Normalizations of the Chlamydomonas transcriptome dataset. (Supports Figure 1).
(A) Normalization steps applied to our datasets and corresponding RNAseq namesakes.
(B) Distribution of expression estimates from 518 RNAseq samples in the original dataset and after each normalization step.

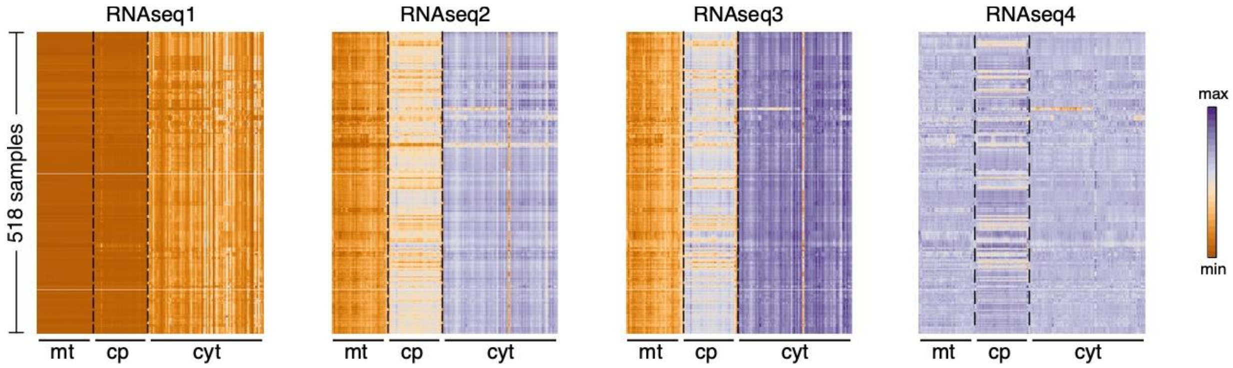

Supplemental Figure 2. How ribosomal protein genes (*RPG*s) respond to each normalization step. (Supports Figure 1).
Expression estimates for 78 cytosolic (cyt), 38 mitochondrial (mt) and 37 plastid (cp) *RPG*s were extracted from each RNAseq dataset (listed above each heatmap). The heatmaps themselves were not normalized: the highest expression value is shown in dark purple, and lowest in gold.
Note how cytosolic *RPG*s appear more highly expressed than either mitochondrial or plastid *RPG*s in RNAseq1-3. However, the last normalization to mean brings all *RPG*s on similar scales regardless of the final localization of their gene products.

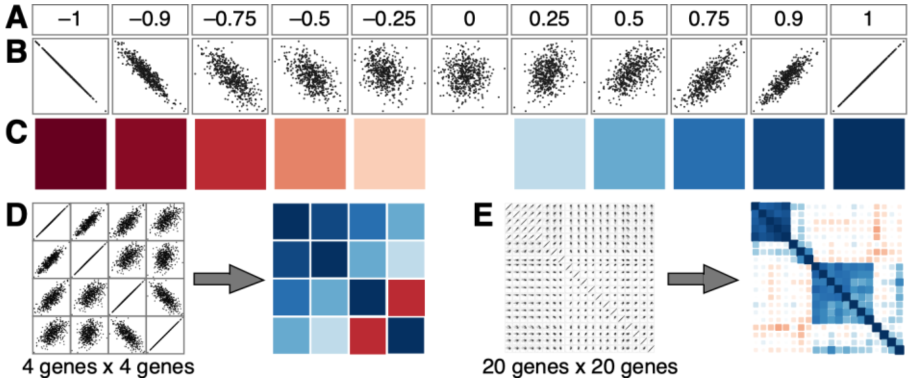

Supplemental Figure 3. The R package *corrplot* and visualization of large correlation matrices. (Supports Figure 1).
Pearson’s correlation coefficient values **(A)** derived from the scatterplots of 518 expression value estimates for two genes **(B)** are converted to colored squares **(C)** ranging from deep red (for values close to -1) to dark blue (for values close to +1).
Example of a 4 x 4 gene matrix **(D)** and 20 x 20 gene matrix **(E)** visualized as scatterplots or as a correlation matrix drawn in *corrplot*.

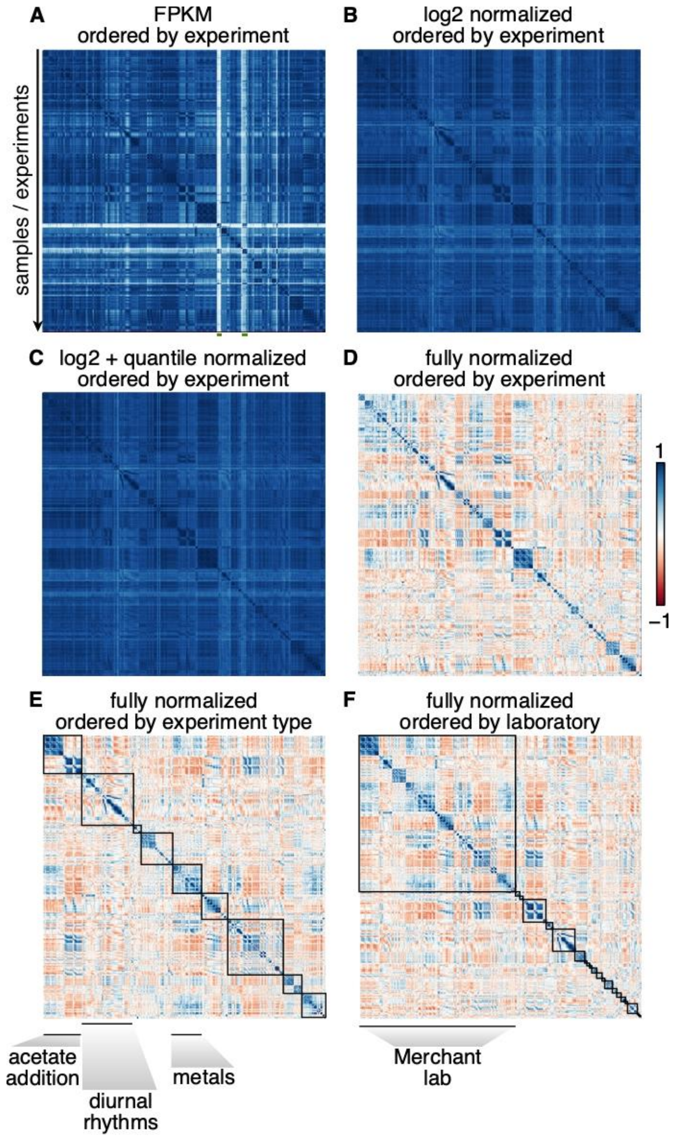

Supplemental Figure 4. Correlations between experimental samples and normalization methods. (Supports Figure 1).

Pearson's correlation coefficients between samples were calculated by using expression estimates from all nuclear genes from RNAseq datasets RNAseq1 (with FPKM values) (**A**), RNAseq2 (log_2-normalized) (**B**), RNAseq3 (log_2- and quantile-normalized) (**C**) or RNAseq4 (log_2-, quantile- and normalized to mean) (**D**), all ordered by experiment. The green lines in panel (**A)** indicate the position of samples collected over the Chlamydomonas sexual cycle. In panels (**E**) and (**F**), experiments were reordered by type of experiment (**E**) or by laboratory of origin (**F**), each indicated by the squares around samples. Over half of all samples were generated by the Merchant laboratory, as shown in (**F**). Experiment types: acetate addition; diurnal rhythms; light and photoperiod; metabolism; metals; mutants; nitrogen; signaling; stress.

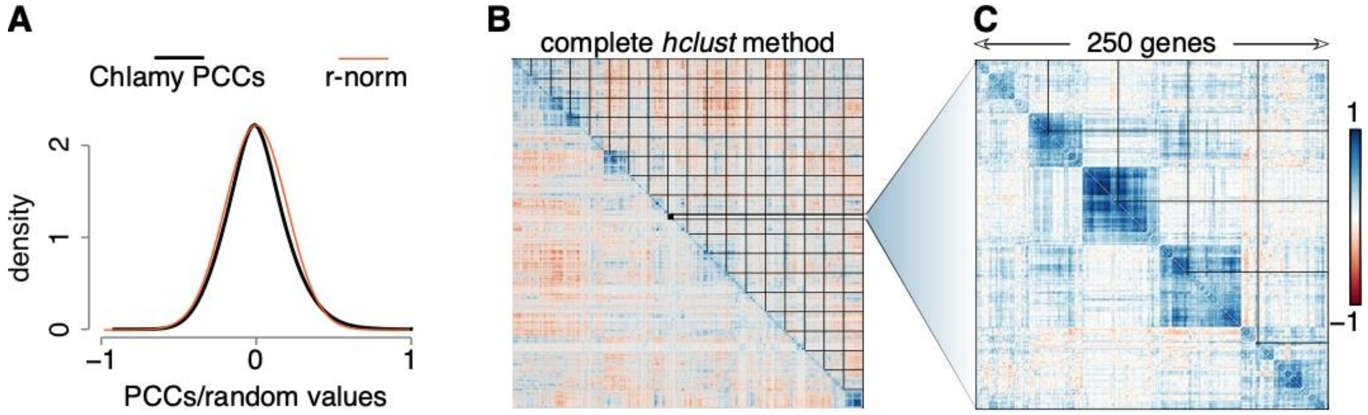

Supplemental Figure 5. Chlamydomonas gene pairs are largely not co-expressed. (Supports Figure 1).
(A) Normal distribution of pairwise Pearson's correlation coefficients (PCCs) of gene expression estimates between Chlamydomonas genes.
(B) Genes clustered based on hierarchical clustering show strong co-expression
(C) Correlation matrix for genes 8,001 to 8,250 (numbers based on their order after clustering).
The grid in the upper right triangle indicates the size of a 1,000x1,000 **(B)** or 50x50 **(C)** gene matrix, respectively.

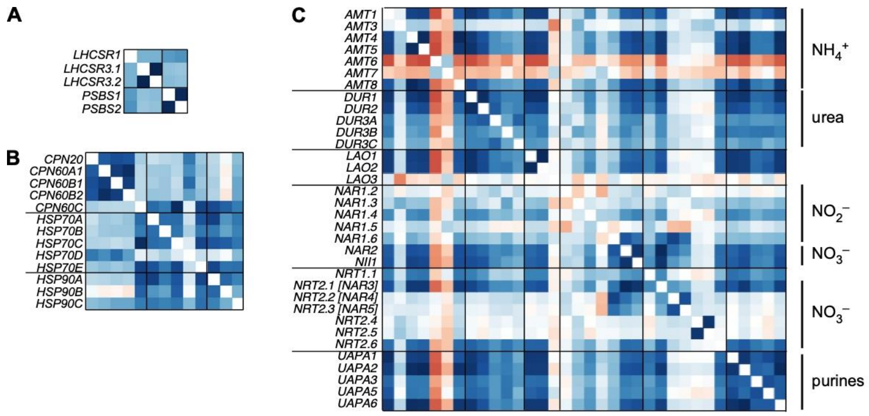

Supplemental Figure 6. Testing known patterns of co-expression in the RNAseq4 data set. (Supports Figures 2-4).
(A) Correlation matrix between the high light-induced genes *LHCSR1*, *LHCSR3.1*, *LCHSR3.2*, *PSBS1* and *PSBS2* using the RNAseq4 data set.
(B) Correlation matrix between chaperonin genes (*CPN20*, *CPN60*) and heat shock protein genes (*HSP70*, *HSP90*) using the RNAseq4 data set.
(C) Correlation matrix between genes related to nitrogen transport and assimilation using the RNAseq4 data set.

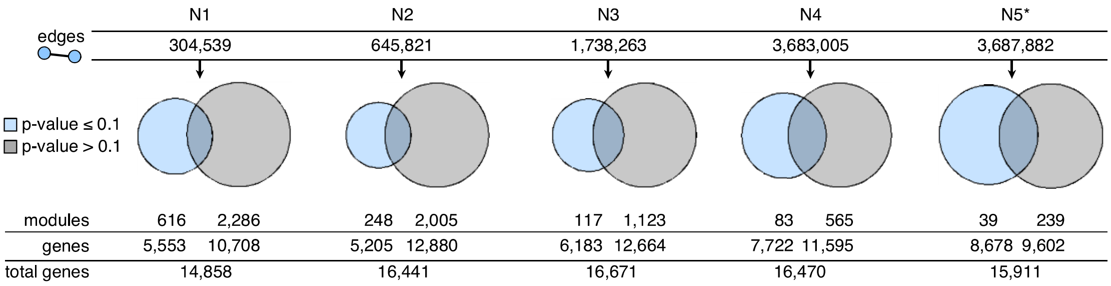

Supplemental Figure 7. From co-expression cohorts to co-expression modules. (Supports Figures 3 and 6).
Summary of the number of edges for each network used as input for the detection of co-expression modules.
We then applied a significance cut-off of 0.1 to identify highly significant co-expression modules (shown in light blue). We lastly counted the number of nuclear genes represented by co-expression modules for each network. The N3 network offers a good compromise between module size (see also Supplemental Table 2).

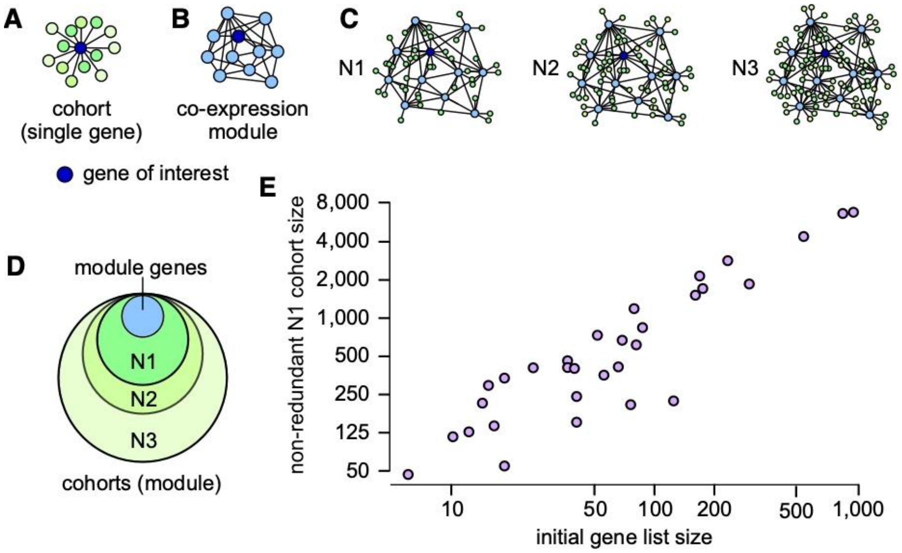

Supplemental Figure 8. Using module nodes as baits to identify co-expressed genes. (Supports Figure 6).

(A) The cohort of genes co-expressed with a given gene (blue circle at the center) is a function of the network, which restricts the size of the cohort based on edge lengths (represented here by lines of various length linking the center gene to its co-expressed cohorts (shown as shades of green, the darker green for network N1, and the lightest green for network N3).

(B) A representative co-expression module, composed of highly connected hub genes.

(C) A more realistic representation of co-expression modules at each network stringency level: hub genes are connected to their co-expressed cohorts, only some of which already belong to the module.

(D) Genes that belong to a given module and their co-expressed cohorts, as nested circles (proportional to the number of constituent genes).

(E) Scatterplot representation of cohort sizes from network N1 associated with user-defined gene lists. Since genes from the initial list are likely to be co-expressed with a subset of the same genes, we reduced cohort size to unique genes by applying the unique() function in R.

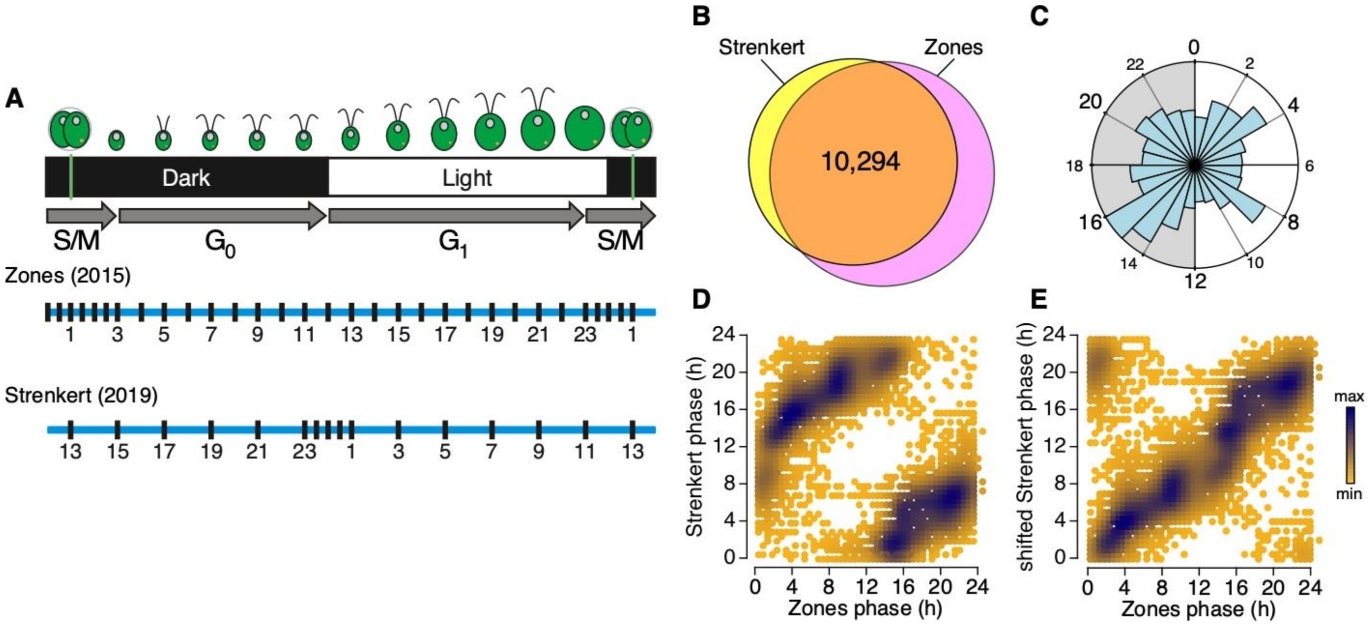

Supplemental Figure 9. Convergence of diurnal phase between two time-courses. (Supports Figure 7 and 8).
(A) Schematic representation of collected samples over the Chlamydomonas diurnal cycle in Zones et al., (2015) and Strenkert et al., (2019).
(B) Overlap between the number of nuclear genes showing a diurnal expression pattern in each study. The overlap in the Venn diagram comprises 10,294 genes.
(C) Distribution of diurnal phases for the high-confidence diurnally rhythmic genes identified in (**B**).
(D, E) Scatterplot of diurnal phases, as determined by Zones et al., (x-axis) and Strenkert et al., (y-axis), before **(D)** and after **(E)** correcting for the 12 h phase shift in the original datasets (Zones et al. used dawn as time 0, while Strenkert et al. used dusk).

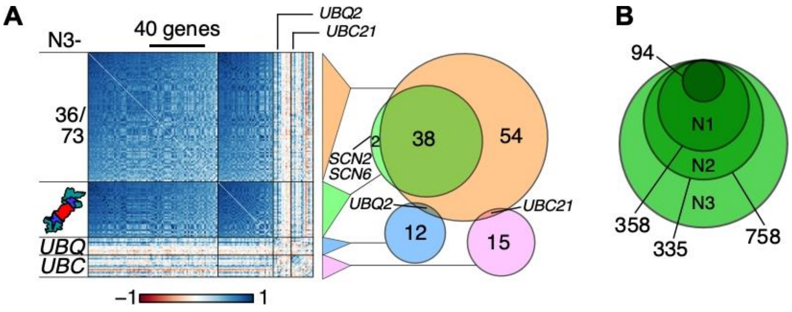

Supplemental Figure 10. Co-expression of the protein degradation machinery is limited to the 26S proteasome. (Supports Figure 6).

(A) Correlation matrix of protein degradation modules N3-36 and N3-73 against genes encoding components of the 26S proteasome (shown as a cartoon), ubiquitin (*UBQ*) and ubiquitin conjugating enzymes (*UBC*). The extent of overlap between each gene list is shown on the right.

(B) Co-expressed cohorts, shown as nested Venn diagrams, associated with genes from protein degradation modules from networks N1-N3.

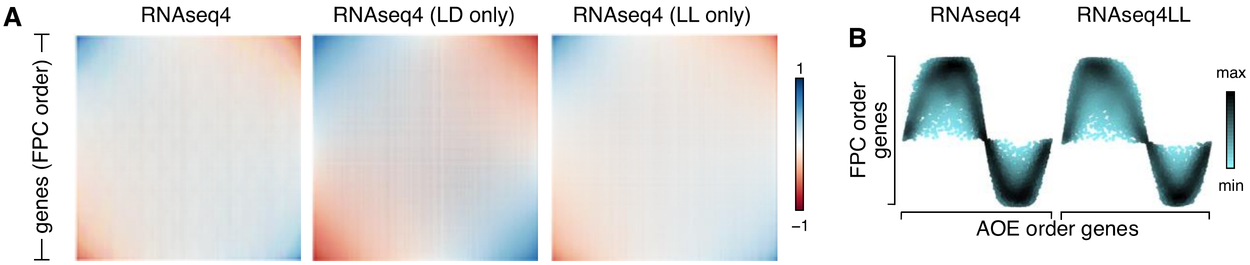

Supplemental Figure 11. Genes Cluster Based on their Diurnal Phase. (Supports Figure 8).

(A) Co-expression matrix of the 17,741 Chlamydomonas nuclear genes, ordered based on clustering by the First Principle Component (FPC) method built into *corrplot*, using RNAseq4, RNAseq4LD and RNAseq4LL as input.

(B) Scatterplot of diurnal phases from 10,294 high-confidence diurnally rhythmic genes, ordered based on the FPC clustering method (y axis) or Angle of the Eigenvector (AOE) clustering method (x-axis) built into *corrplot*, for RNAseq4 and RNAseq4LL.

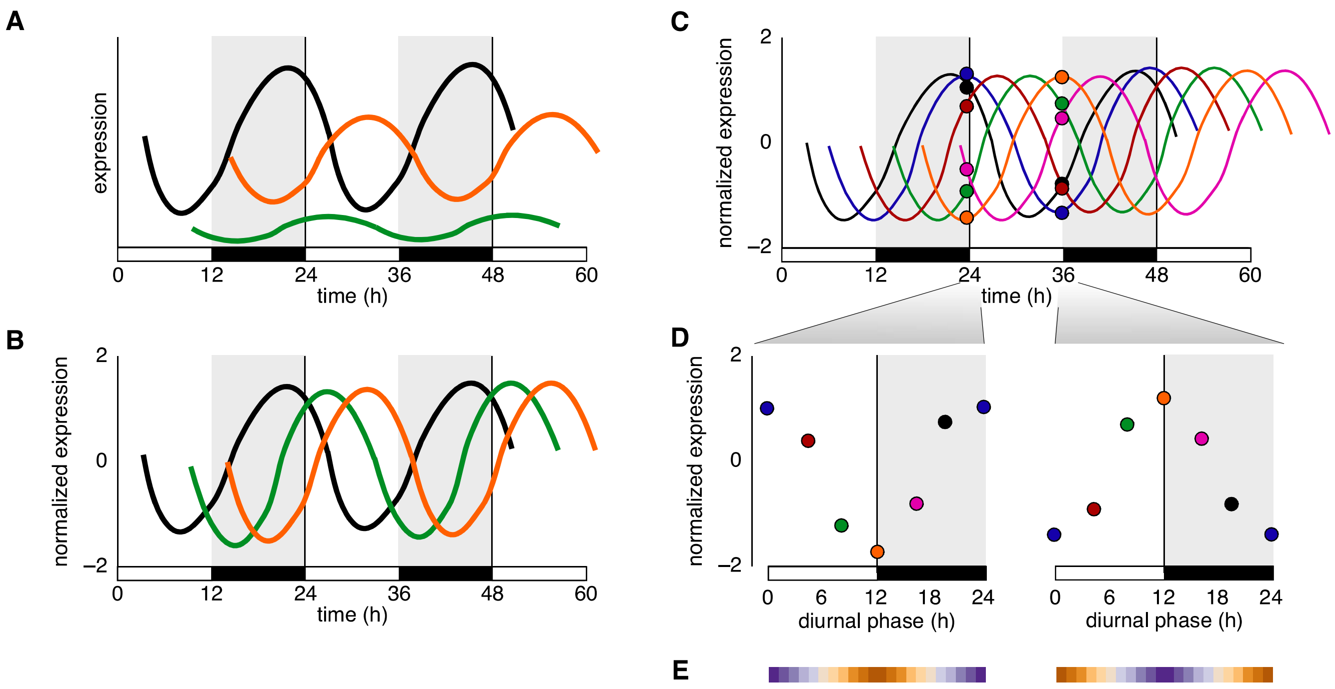

Supplemental Figure 12. Molecular timetable method to extract diurnal information from single time-points. (Supports Figure 9)

(A) Diurnal expression profile of three genes with distinct amplitude and mean expression levels.

(B) Same genes as in (A), but after Z-score normalization to bring all genes onto a comparable scale.

(C) Six genes with representative diurnal phases (each gene peaking 4 h after the last). Circles indicate the minimum and maximum normalized expression level for each gene.

(D) For a single time-point sample collected at dawn (left panel), the normalized expression level of each representative gene is plotted as a function of the gene's known phase during a typical diurnal experiment (along the x-axis). The right panel shows the same analysis carried out for a single time-point collected at dusk, this 12 h out of phase with the left-side panel. Note how the normalized expression levels of the representative genes form a wave with a peak corresponding to the collection time.

(E) Turning normalized expression data from (D) into a heatmap (lowest values in orange, highest values in purple).

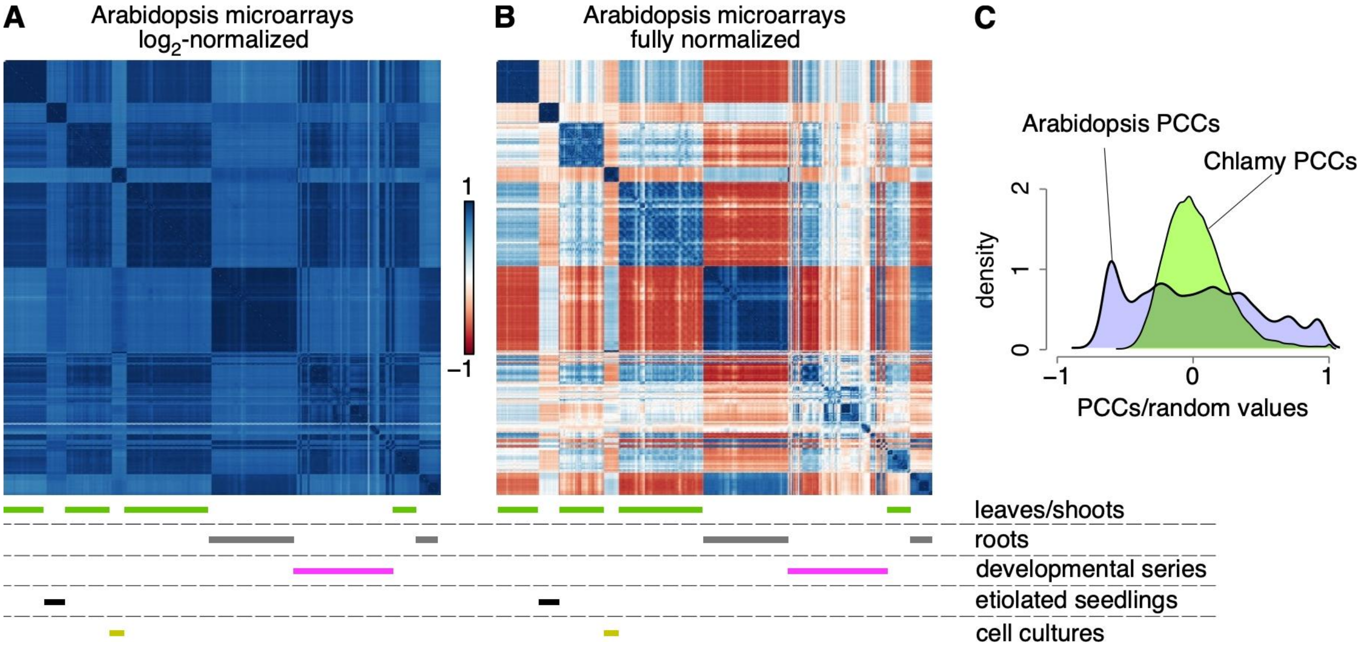

Supplemental Figure 13. Arabidopsis microarray data clearly differentiates between tissue types. (Supports Figure 1).

(A) Correlation matrix showing the correlation between 350 microarray experiments, based on log_2-normalized expression data.

(B) Correlation matrix for the same 350 microarray experiments, after full normalization. For (**A**) and (**B**), the tissues corresponding to each sets of microarrays are indicated below the matrix.

(C) Distribution of pairwise PCCs between Arabidopsis experiments (purple) and Chlamydomonas samples (green).

Supplemental Table 1. Summary of expression estimates across all conditions and samples. The number of genes is given, with the corresponding percentage of the nuclear genome shown in parentheses.

	Expression cut-offs				
Number of instances	FPKM>1	FPKM>5	FPKM>10	FPKM>50	FPKM>100
0-50	2,447 (13.8%)	6,574 (37.1%)	10,046 (56.6%)	15,559 (87.7%)	16,605 (93.6%)
51-100	634 (3.6%)	1,645 (9.3%)	1,383 (7.8%)	494 (2.8%)	264 (1.5%)
101-150	562 (3.2%)	1,037 (5.8%)	878 (4.9%)	327 (1.8%)	129 (0.7%)
151-200	551 (3.1%)	864 (4.9%)	652 (3.7%)	204 (1.1%)	100 (0.6%)
201-250	621 (3.5%)	732 (4.1%)	549 (3.1%)	163 (0.9%)	73 (0.4%)
251-300	638 (3.6%)	750 (4.2%)	514 (2.9%)	110 (0.6%)	69 (0.4%)
301-350	708 (4.0%)	710 (4.0%)	477 (2.7%)	137 (0.8%)	80 (0.5%)
351-400	894 (5.0%)	750 (4.2%)	528 (3.0%)	125 (0.7%)	97 (0.5%)
401-450	1,404 (7.9%)	960 (5.4%)	697 (3.9%)	155 (0.9%)	84 (0.5%)
451-500	3,298 (18.6%)	1,846 (10.4%)	1,033 (5.8%)	250 (1.4%)	104 (0.6%)
501-518	5,955 (33.6%)	1,812 (10.2%)	929 (5.2%)	197 (1.1%)	121 (0.7%)
	FPKM<1	FPKM<5	FPKM<10	FPKM<50	FPKM<100
518	494 (2.8%)	1,877 (10.6%)	3,737 (21.1%)	10,731 (60.5%)	13,224 (74.5%)

Supplemental Table 2. Cohort and modules sizes for co-expression data derived from the RNAseq4 dataset.

	network	mean	min	max
cohorts	N1	17	1	68
	N2	36	2	125
	N3	98	10	285
	N4	208	26	557
	N5	438	66	1,004
modules (p-value < 0.1)	N1	12	3	29
	N2	28	3	56
	N3	86	8	143
	N4	194	51	340
	N5	349	53	779
modules (p-value > 0.1)	N1	7	3	22
	N2	11	3	39
	N3	27	3	92
	N4	69	3	226
	N5	156	3	405

Supplemental Table 3. Summary of GO terms enriched in N3 co-expressed modules.

cluster	GO category	Enrichment	p-value
Cell division and DNA replication			
N3-1	Cell division	>100	1.1×10^{-04}
	DNA replication	58	1.9×10^{-05}
	Organelle organization	16	8.4×10^{-05}
N3-41	DNA replication	>100	3.2×10^{-09}
	DNA metabolic process	39	4.0×10^{-05}
N3-53	DNA replication	>100	1.7×10^{-15}
	DNA metabolic process	24	2.3×10^{-04}
N3-67	DNA replication	>100	2.2×10^{-07}
	DNA metabolic process	59	8.3×10^{-06}
N3-117	DNA replication	>100	4.6×10^{-15}
Transcription			
N3-100	Transcription by RNA pol III	>100	3.7×10^{-05}
N3-104	Transcription by RNA pol III	>100	2.3×10^{-05}
Translation			
N3-5	Ribosome biogenesis	86	5.0×10^{-06}
N3-7	Ribosome biogenesis	46	3.9×10^{-05}
	RNA metabolic process	18	5.3×10^{-08}
N3-76	RNA metabolic process	14	1.5×10^{-05}
N3-94	rRNA processing	94	4.4×10^{-06}
N3-61	Translation	57	2.4×10^{-46}
N3-109	Translation	62	1.3×10^{-40}
N3-13	tRNA aminoacylation for protein translation	92	5.2×10^{-06}
	Response to cadmium	90	2.5×10^{-04}
	Amino acid biosynthetic process	61	5.3×10^{-07}
N3-19	Regulation of translation initiation	>100	5.2×10^{-06}
	Ribonucleoprotein complex assembly	>100	1.9×10^{-10}
	tRNA aminoacylation for protein translation	76	9.6×10^{-06}
Protein degradation			
N3-36	proteasomal protein catabolic process	93	2.7×10^{-04}
N3-73	proteasomal protein catabolic process	79	3.8×10^{-04}
Photosynthesis			
N3-18	protoporphyrinogen IX biosynthetic process	>100	1.4×10^{-09}
N3-35	protoporphyrinogen IX biosynthetic process	>100	4.6×10^{-10}
N3-48	photosynthetic electron transport in photosystem I	>100	2.2×10^{-05}
	ATP synthesis coupled proton transport	>100	1.8×10^{-04}
N3-52	Photosynthesis, light harvesting	>100	1.4×10^{-42}
	Protein-chromophore linkage	>100	3.8×10^{-42}
Cilium assembly			
N3-23	Cilium assembly and movement	>100	2.5×10^{-08}
	Intraciliary transport	>100	3.2×10^{-05}
N3-64	Microtubule-based process	58	1.6×10^{-05}
N3-108	Intraciliary transport	>100	3.5×10^{-06}
	Cilium assembly	>100	4.8×10^{-05}
N3-116	Motile cilium assembly	>100	1.3×10^{-07}
	Intraciliary transport	>100	4.2×10^{-05}

Enrichment is defined as the number of genes with a given GO term, divided by the total number of genes with this GO term in the genome.

Supplemental Table 3 (cont). Summary of GO terms enriched in N3 co-expressed modules.

cluster	GO category	Enrichment	p-value
Metabolism			
N3-44	Mitochondrial electron transport	>100	2.8×10^{-05}
	ATP synthesis coupled proton transport	>100	1.2×10^{-13}
N3-63	Cysteine biosynthetic process (from serine)	>100	1.2×10^{-05}
N3-87	Glycine metabolic process	>100	1.6×10^{-05}
N3-89	TCA cycle	>100	7.4×10^{-16}
	Acetyl-CoA biosynthetic process	>100	1.1×10^{-05}
Transport			
N3-27	Vesicle-mediated transport (Golgi to ER)	>100	8.1×10^{-05}
N3-51	Vesicle-mediated transport	22	3.0×10^{-05}
	Protein transport	21	3.8×10^{-05}
N3-70	Golgi vesicle transport	66	1.6×10^{-05}
N3-59	Phosphate ion transport	>100	5.9×10^{-07}
N3-2	Ammonium transport	>100	1.1×10^{-07}
N3-90	Ammonium transmembrane transport	>100	2.9×10^{-08}
N3-84	Iron ion homeostasis	>100	2.7×10^{-06}
	Iron ion transport	>100	3.4×10^{-09}
N3-20	Copper ion transport	>100	2.7×10^{-06}
Other			
N3-3	Response to hormone (cytokinin)	69	4.8×10^{-04}

Enrichment is defined as the number of genes with a given GO term, divided by the total number of genes with this GO term in the genome.
