# Supplementary material for: Co-expression networks in Chlamydomonas reveal significant rhythmicity in batch cultures and empower gene function discovery
Source: Plant Cell. 2021 Feb 2;33(4):1058–82. doi: 10.1093/plcell/koab042 (PMC8226298; doi:10.1093/plcell/koab042)
Supplement: koab042_Supplementary_Data [file tpc.00822.2020-s04.pdf › tpc.00822.2020-s04.pdf]

Co-Expression Networks in Chlamydomonas Reveal Significant Rhythmicity in Batch Cultures and Empower Gene Function Discovery

Patrice A. Salomé and Sabeeha S. Merchant

Corresponding author: Patrice A. Salomé salome@chem.ucla.edu

Review timeline:

**TPC2020-RA-00822**	Submission received:	Oct. 05, 2020
	1st Decision:	Nov. 12, 2020 *request revisions*
**TPC2020-RA-00822R1**	1st Revision received:	Jan. 15, 2021
	2nd Decision:	Jan. 19, 2021 *accept*
	Final acceptance:	Jan. 25, 2021

REPORT: (The report shows the major requests for revision and author responses. Minor comments for revision and miscellaneous correspondence are not included. The original format may not be reflected in this compilation, but the reviewer comments and author responses are not edited, except to correct minor typographical or spelling errors that could be a source of ambiguity.)

TPC2020-RA-00691 1st Editorial decision – *request revisions* **Nov. 12, 2020**

We have received reviews of your manuscript entitled "Co-Expression Networks in the Green Alga *Chlamydomonas reinhardtii* Empower Gene Discovery and Functional Exploration." Thank you for submitting your best work to The Plant Cell. The editorial board agrees that the work you describe is substantive, falls within the scope of the journal, and may become acceptable for publication, pending revision and potential re-review.

Please note the following in addition to addressing all reviewer comments:

+The reviews are relatively short; however the reviewers emphasized in the post-review consultation that a substantial rewrite (although no additional experimental data) is needed. Reviewer 1 in particular found the manuscript difficult to follow and in need of substantial revision to appeal to a wider audience by better focusing on the important results and clarifying many of the methods.

+In post-review consultation, Reviewer 2 added a request for releasing the annotated R-code on GitHub, which "would definitely solve the methods problem and make this manuscript outlast its publication date" (and this might not be a substantial amount of code as it seems corrplot and Cytoscape were used to generate most of the cross correlations)..

TPC2020-RA-00822R1 1st Revision received **Jan. 15, 2021**

--- Editors and Reviewer comments (**Response from Authors in blue**):

We thank the editors and reviewers for their helpful comments. We were pleased with their enthusiastic opinion of our work, and appreciate their comments in helping us improve the manuscript.

Editors (Comments for the Author):

+The reviews are relatively short; however the reviewers emphasized in the post-review consultation that a substantial rewrite (although no additional experimental data) is needed. Reviewer 1 in particular found the manuscript difficult to follow and in need of substantial revision to appeal to a wider audience by better focusing on the important results and clarifying many of the methods.

RESPONSE: We have greatly expanded the methods in the revised manuscript. We have also modified the abstract, rewrote the entire introduction to focus on the question at hand (how to prioritize candidate genes for follow-up) and modulated some statements that might have been overly subjective or too effusive early in the results. We would

however like to keep the statement that the correlation pattern observed between diurnal samples (Figure 2E) IS striking, because we have not seen anything quite like this before.

We have removed the section on ferredoxins, for space. Other sections do touch on using co-expression to differentiate between members of a gene family. We hope that the changes to the text have improved the manuscript.

+In post-review consultation, Reviewer 2 added a request for releasing the annotated R-code on GitHub, which "would definitely solve the methods problem and make this manuscript outlast its publication date" (and this might not be a substantial amount of code as it seems *corrplot* and cytoscape were used to generate most of the cross correlations).

RESPONSE: We now provide the scripts used for analyses in a series of Supplemental protocols. The scripts can surely be improved upon, but they will work.

While assembling these protocols, we noticed that the Methods section "Identification of Co-Expression Cohorts" was misleading, as it talked about extracting genes that were co-expressed with each module, when in fact it should have been about the extraction of genes co-expressed with any gene list. This has now been corrected and expanded to mention our handling of community-curated gene lists, which, unlike genes from co-expressed modules, are not necessarily all co-expressed. For example, Figure 5A, "genetics list", consists of genes identified from a screen looking for Chlamydomonas mutants with defects in the cell cycle. Only a subset of these genes is co-expressed, and extracting the co-expression cohort of the entire list would dilute the overall co-expression and make analysis more difficult. We therefore decided to implement a cutoff for co-expression, to restrict these data sets to highly co-expressed genes. A detailed set of Supplemental Protocols is provided.

+Can the step-by-step guide be a supplemental protocol? It was not clear what parts of the methods were developed by the authors: the cover letter suggests the Wisecaver 2017 TPC paper was key, but the manuscript doesn't clearly state what methods were taken or adapted from Wisecaver et al. (or other papers cited) and what was newly developed. Wisecaver et al. similarly constructed MR-based networks and used ClusterONE to assign modules, for example.

RESPONSE: This project has spanned several years. In its initial stages, the data set used comprised of non-normalized RNA-seq data, which succeeded in identifying co-expression relationships between genes. With the publication of the Wisecaver 2017 TPC paper however, we quickly realized that normalization would be key to extracting more meaningful information.

Importantly though, the Wisecaver paper and the AICOdb paper do NOT provide any code for normalization, as the expression data sets were downloaded from online databases (Wisecaver) or generated by the authors without releasing the code (AICOdb). Same for the mutual rank values later used to generate co-expression networks in Cytoscape. Thus, we followed the normalization steps described for AICOdb but came up with our own code, now provided as Supplemental Protocols.

ClusterONE in Cytoscape uses lists of genes and their co-expressed genes to identify co-expression modules. Any and all code for this step is built in to ClusterONE, whose output is a list of modules with a p-value (if the file is saved as a .csv file. For the longest time, we did nothing with the clusters because we were not able to save the p-values).

-- Reviewer comments:

Reviewer #1 (Comments for the Author):

There are several aspects of the co-expression network analysis of Chlamydomonas presented in this manuscript that I think are important, timely, and will be of great interest to the larger plant biology community. The exploration of negative correlations was novel and interesting. I also appreciated the results regarding the importance of considering batch effects and diurnal rhythmicity when constructing and analyzing gene networks.

Point 1. However, the methods provided are not sufficient for one to replicate the work, the results section is difficult to follow in places and generally reads like a chronicle of all the bioinformatic analyses tried crammed into one manuscript. I think that the paper would really benefit from a careful rewrite to emphasize the novel aspects of the paper and trim other parts.

RESPONSE: We have expanded the methods section and provided additional scripts to help the reader run their own analyses their favorite genes and gene lists.

This manuscript is admittedly more of a collection of vignettes illustrating what can be done with co-expression analyses in Chlamydomonas and beyond. Ideally, we would have loved to characterize 100-200 insertion mutants in Chlamydomonas for a chosen phenotype, based on the co-expression profile of their affected genes. One obvious

set of genes would have been those involved in flagella biogenesis or function. However, 1) this is not a focus of the laboratory, and would require an outside collaboration, since the first author has since left Academia, and 2) this experiment would be expensive, since each insertional mutant would cost $100 from the Chlamydomonas Resource Center.

Thus, rather than focusing on one pathway, we have decided to make the data sets available to the community, so that others can run their own analyses to find new genes with roles in their favorite pathways. This approach is likely to be complementary to genome-wide insertional screens being performed in Chlamydomonas.

Point 2. Given some of the interesting results, the Title and Short Title are nondescript. For example, the results summarized in at the end of the abstract lines 46-50 are the novel aspects of this paper that I think could be emphasized better in the title and elsewhere.

RESPONSE: The long and short titles are tricky to distill the contents of the manuscript into a few words. We agree that generating lists of high-confidence candidate genes for follow-up studies and the fact that bulk RNA-seq samples retain significant rhythmicity are the main conclusions, but they are complicatedly unrelated to one another and would make for an awkward long title.

We offer a few suggestions in the title page of the manuscript, but would greatly appreciate suggestions from the editors.

Point 3. the Introduction is odd. It starts out with a review of the history of genetics, which I think is overly broad for this analysis. There is some weird/distracting use of terminology such as 'deep' sequencing as well as 'comparative mode' (ln 87) instead of differential expression. The authors acknowledge that other work on coexpression networks in Chlamydomonas have been done. Line 100 says that the major limitation of prior analyses is the fact that they are focused on single genes or offer precomputed modules, but isn't this what is being done in the current paper as well? I'm not sure why not providing 'a visual summary of underlying correlations' is a limitation of prior work either, because they do provide the underlying global network for anyone to visualize as they please. I don't see how the current analysis is any more searchable than the prior analyses either. The authors don't provide an interactive web tool for users to run their corrplot code to make new modules. I would instead focus the introduction to setup their interesting results as well (eg why are negative correlations important gene associations to include in global networks).

RESPONSE: We have changed the word "comparative" to "contrasting", which, as explained in the rest of the sentence, we mean as "by comparing the wild type to the mutant, or between untreated and treated cultures". In Arabidopsis for example, the iron deficiency-induced transcription factor FIT1 was identified this way: by comparing the transcriptional profile of wild-type roots collected from iron-replete and iron-limited plants, and looking at transcription factor genes that were induced under low iron conditions.

We have also significantly modified the introduction to focus on integration of multiple approaches (mutant screens, proteomics, transcriptome profiling) to prioritize candidate genes for follow-up studies. We have removed the passage corresponding to line 100; we realize that the type of platform used is a question of personal preference. We tend to not like network graphs with genes (indicated by bubbles) connected by edges, and much prefer the graphical summary provided by *corrplot*. We also feel like we have little control over parameters in other platforms, but have total control over our method. Finally, our method is portable and can be run at any time from a laptop computer with the simple input of a gene list.

RNA-seq is short for transcriptome deep sequencing, so we think that one "deep" should remain.

Negative correlations cannot be included in a co-expression network. For example, all the co-expression modules we obtained with ClusterONE show very strong positive correlations among their constituent genes; importantly, none show anti-correlation because the input files for ClusterONE have already selected only the genes with the highest co-expression potential. We had naively thought of using anti-correlated genes to generate anti-correlation networks, but we had to satisfy ourselves with anti-correlation cohorts (that is, gene X is anti-correlated with a suite of genes). Simply put, anti-correlation requires a gene to be anti-correlated relative to.

Point 4. The results are very difficult to follow at times (eg analyses of RPGs in Arabidopsis and *Physcomitrella* lines 404 - 416, and the transcription factors in Arabidopsis lines 444-457 are particularly difficult to follow). I wasn't always sure why an analysis was being done. I really didn't follow the last part about transferring mean Chlamydomonas expression over to *Volvox* and *Chromochloris* orthologs. Why not perform the analysis again with the full *Volvox* and *Chromochloris* gene sets?

RESPONSE: +*RPG*s. We thank the reviewer for pointing to the absence of the gene list for Arabidopsis *RPG*s (point 11 below), now provided as a Supplemental Data Set. We have also added a section in Methods about the analysis of Arabidopsis *RPG*s and have completely rewritten the section in results.

We also discovered that one of the gene sets labeled as "unclear" in the Figure was actually a duplicated copy of the plastid *RPG*s that snuck into the master file. We have now rerun the analysis and have modified the Figure accordingly. The main conclusions are not affected, although now the "unclear" category behaves as a single group.

We have removed the mention to *Physomitrium* (*Physcomitrella*) *patens RPG*s, since we did not show the results in a figure.

Transcription factors. We have removed this part of results, to focus the text on the more positive aspects of our analyses. We had hoped to identify direct targets of transcription factors based on their co-expression cohorts, but this was probably too optimistic.

Transferring Chlamydomonas data to *Volvox* and *Chromochloris*. The beginning of the paragraph did mention that neither algae have a high-density diurnal time course, which is critical to determine the mean expression of a given gene, its associated standard deviation and the predicted diurnal phase. Since this information is not currently available for either algae, it is not possible to use the full *Volvox* and *Chromochloris* gene sets, and are left with putative orthologs between Chlamydomonas and *Volvox* (or *Chromochloris*). We have made this point clearer in the text.

Point 5. Many of the methods are unclear. For example, I don't understand how the co-expression cohorts were called. I also couldn't find methods text on the molecular timetable approach or the analysis in other green algae. A lot of methods text is presented in the results section instead of the methods section.

RESPONSE: We apologize for the abridged Methods provided. We have since expanded this section to cover the calling of co-expression cohorts and the application of the molecular timetable method. The methods are also supported by the Supplemental Protocols.

We would like to keep much of the "methods text" in the main text, because it is not our intention for the reader to go back and forth between results and methods. We realize that some of the analyses we performed are not widely used, thus placing the onus on us, the authors, to explain as we walk the reader through the results. In the scRNA-seq manuscript, we similarly walked the reader through dimensionality reduction because this method might not be known to most readers (or in fact to the reviewers), while other readers familiar with the technique will likely skip over that part.

Point 6. Line 252. This is not true. There is the potential for genes with low variance to appear correlated with other genes with low variance, illustrating the importance of excluding genes with low/no variance from the network (see Usadel 2009 Plant Cell & Environ).

RESPONSE: We have deleted the sentence in the revised manuscript after looking at the Usadel review, as we believe that our previous statement does not apply to the normalized data sets we used.

Most analyses of co-expression make use of data that are normalized via GCRMA or equivalent followed by $\log_2$ transformation. For example, all normalized expression values from ATTED-II are $\log_2$-transformed. The GCRMA normalization step is similar to the quantile normalization step we included in our pipeline, as per AlCOdb. The next normalization step in our pipeline (centering to the mean of each gene) is however not part of ATTED-II (but it is in AlCOdb, which uses mutual ranks to define co-expression) and is akin to a Z-score normalization. In fact, normalization of the RNAseq3 data set (after quantile normalization) by either the mean or the standard deviation of a gene results in the exact same PCC values. Our normalized expression values follow a normal distribution, which the starting RNA-seq (or microarray) data sets did not accomplish.

Importantly, the final data set we used to calculate PCCs, RNAseq4, is dimensionless and brings all genes to the same range. Therefore, genes with low variance will be on the same scale as any other gene. While we might run the risk of overinflating their variation by keeping these low-variance genes, we decided it was safer to keep them rather than removing a subset of genes based on arbitrary expression cut-offs that, when looking at over 500 independent samples, would have led to the removal of thousands of genes (as illustrated in Supplemental Table 1).

Reviewer #2 (Comments for the Author):

This manuscript by Salomé and Merchant is a large effort to reanalyze existing RNAseq data sets from Chlamydomonas reinhardtii to determine a holistic understanding of gene coexpression modules. The authors use a very large data set to derive deep insights into co-expressed genes. This is a fantastic resource for Chlamydomonas biologists and will greatly expand our understanding of Chlorophycean and other photosynthetic algal biology. This is especially important because the genomes of these organisms are not well understood and these large scale re-analysis and meta experiments will develop insight infrastructure useful to all researchers.

In particular the diurnal experiment cross-correlation with other species, both algal and vascular plant (Arabidopsis) suggests that model organism data will be highly valuable to other related species both closely related and distantly related.

Overall, this paper is a strong candidate for publication in The Plant Cell. I have a few Major and Minor Criticisms to improve the manuscript, but generally, this is a great paper.

Major Criticisms

Point 1. With the analyses of well understood pathways in Chlamydomonas, there is not a sufficient discussion of how the RNAseq data correlates with known gene regulatory and biochemical regulatory points in the pathways. E.g. this was done in Fig 3 with proteome data for the cilia. What about other metabolic pathways where regulation is known? How does the RNA-seq data correlate to presumably post-transcriptionally regulated metabolic pathways.

RESPONSE: We understand the reviewer's point. We expect the entire data set to recapitulate transcriptional patterns from each individual RNA-seq data set that comprises it. Indeed, we see clear co-expression across chlorophyll biosynthetic genes. We now provide a new Supplemental Figure 6 with additional examples that explore correlation of genes with known responses:

+high light responses. The genes *LHCSR3.1*, *LHCSR3.2* and *PSBS* are known to be up-regulated in response to high light stress. We see a strong co-expression signature in our data set.

+heat responses. We tested the co-expression between *HSP70* and *HSP90* genes, and saw good positive correlations, as expected.

+nitrogen responses. The laboratory is working on a more thorough analysis of nitrogen responses in Chlamydomonas, but we do offer clear evidence that the entire RNAseq4 data set recapitulates known responses to nitrogen status, based on the expression profiles of various nitrogen transporters. Not all strains used across the 518 samples are NIT+, which had initially stopped us from looking into more detail.

We hope these examples will be sufficient. As we do not plan to release the RNAseq data for all 518 samples (some are unpublished, but we do not make use of any FPKM expression estimates in the manuscript, aside from Supplemental Table 1, in very broad terms), it was our intent to keep the focus on the data set as a whole, rather than looking at individual experiments to see whether they recapitulate known expression patterns.

We also explored transcriptomics changes in response to metal deficiency by looking for genes that are co-expressed with known transporters and homeostasis factors. We are planning on writing these results separately, but the pandemic has placed all analysis on hold (including the determination of metal contents in a set of 200 Chlamydomonas insertional mutants).

Point 2. Histone genes are not polyadenylated when expressed in Chlamydomonas. Because most RNAseq experiments use polyA mRNA, their expression in resultant libraries is expected to be non-quantitative in representation unless some experiments are ribo-minus or related techniques. Second, most read mapping algorithms multi-map the read, which in highly repetitive regions may lead to errors in quantitation. It appears from the methods that they did multi mapping correction. Thus, I have questions about the validity of the histone expression data.

RESPONSE: We appreciate the reviewer's concerns. The non-normalized RNA-seq data set did show signs that certain experiments (samples collected over the sexual cycle and/or ribodepleted) showed a weaker overall correlation with other samples (as can be seen in Supplemental Figure 4A, as the lighter blue lines in the correlation matrix).

Regardless, we have gone back to the original data and removed samples that were ribodepleted or with histone expression >5FPKM (based on an inspection of mean histone expression levels across all samples) and moved them into a separate data set. We then reran all normalization steps and tested the correlation between all histones in each of the data sets. As shown in the revised Figure 5, replication histone genes remain very highly positively correlated

when focusing on ribodepleted samples, which is consistent with our previous results (Strenkert et al.., 2019). Non-emergency histones (which ARE polyadenylated and are more constantly expressed than cell division-associated histones) are equally correlated across polyA-selected and ribodepleted samples. We do believe that the significant correlation observed for replication histones in polyA-selected samples (~0.5) is likely overinflated due to their very low expression levels in these samples and the normalization to the mean. However, they are clearly strongly correlated in ribodepleted samples. We thank the reviewer for alerting us to the possible misinterpretation of the histone results we presented earlier, and hope that the revised figure and corresponding text appropriately addresses their concerns.

We also think that the next portion of the analysis of histones is the more exciting, and we only noticed it while looking at the co-expression of histone genes in Chlamydomonas. Indeed, all Chlamydomonas histone genes are present as divergent gene pairs in the genome, with no exception (cell division-related and non-emergency histones alike). We think this result is rather striking and deserves a mention here so as not to be lost.

And while we do not understand how all Chlamydomonas histone genes show such a pattern, this is not unique to this alga, as we identified similar (although nothing as drastic) arrangements of histone genes in *Volvox carteri*, *Dunaliella salina*, *Chromochloris zofingiensis* and *Micromonas*. By contrast, neither *Physcomitrium* (*Physcomitrella*) *patens*, *Marchantia polymorpha*, nor Arabidopsis, have any remaining trace of this pattern.

As more genomes are sequenced and annotated, we hope that someone will read this result and wonder how widespread this observation is, and when land plants lost it.

I have concerns with the GO and Cytoscape mapping data. In general, the number of genes that map to these programs is quite low compared to other organisms. Obviously, there is not much the authors can do about that fact, but it might be worth pointing this out as a potential limitation and especially a place where open sourcing the analysis for updating as these annotations improve will be important for future work.

RESPONSE: We fully agree that GO annotation in Chlamydomonas is very limited, and a new version of GO for Chlamydomonas is a long ways away. We did have one figure in an earlier version that showed the number of genes with acronyms (indicative of some level of characterization, although by no means exhaustive or mechanistic) and with associated GO terms.

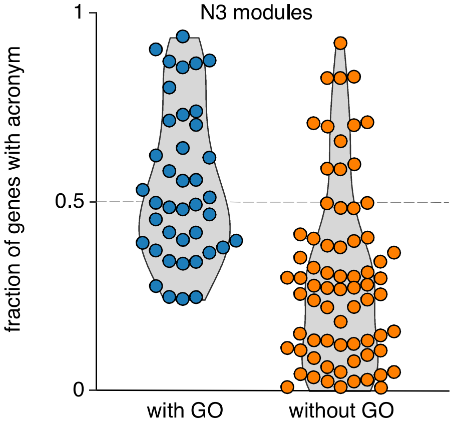

Another figure also showed how the requirement for a gene to have an associated Uniprot ID AND a GO term greatly decreased the size of the gene lists for GO annotation:

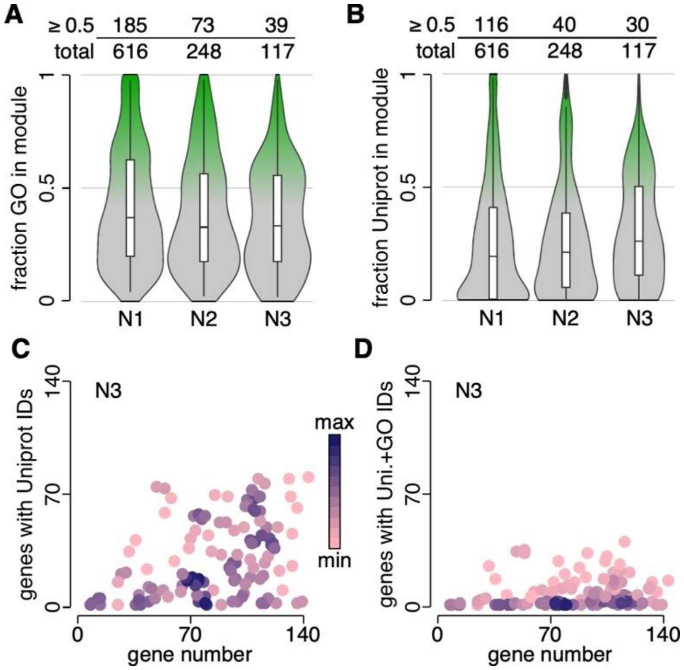

GO annotation in Chlamydomonas reflects the more popular and/or historically important research topics in the alga, including cilia and ribosomes. Since many genes lack annotation, we feel it is best to provide this resource to the community so that any researcher may determine the co-expression cohorts associated with their favorite genes, even when the gene does not belong to one of the co-expression clusters identified here. In fact, the only way to build up the GO catalog of an organism like Chlamydomonas would be to move away from groups with GO terms and characterize unknown genes. The approach we present here can contribute to offering a glimpse at their function, by guilt by association. We admit that the argument is also circular, as genes with GO terms will receive priority.

We will note significant community interest in this approach. We have already contributed co-expression analysis with this dataset for several published studies, and run analyses for other laboratories:

Coexpressed subunits of dual genetic origin define a conserved supercomplex mediating essential protein import into chloroplasts. Ramundo S, Asakura Y, Salomé PA, Strenkert D, Boone M, Mackinder LCM, Takafuji K, Dinc E, Rahire M, Crèvecoeur M, Magneschi L, Schaad O, Hippler M, Jonikas MC, Merchant S, Nakai M, Rochaix JD, Walter P. Proc Natl Acad Sci U S A. 2020 Dec 3:202014294. doi: 10.1073/pnas.2014294117.

Direct visualization of degradation microcompartments at the ER membrane. Albert S, Wietrzynski W, Lee CW, Schaffer M, Beck F, Schuller JM, Salomé PA, Plitzko JM, Baumeister W, Engel BD. Proc Natl Acad Sci U S A. 2020 Jan 14;117(2):1069-1080. doi: 10.1073/pnas.1905641117.

In situ architecture of the algal nuclear pore complex. Mosalaganti S, Kosinski J, Albert S, Schaffer M, Strenkert D, Salomé PA, Merchant SS, Plitzko JM, Baumeister W, Engel BD, Beck M. Nat Commun. 2018 Jun 18;9(1):2361. doi: 10.1038/s41467-018-04739-y.

TPC2020-RA-00822R1 2nd Editorial decision – *accept* **Jan. 19, 2020**

We are pleased to inform you that your paper entitled "Co-Expression Networks in Chlamydomonas Reveal Significant Rhythmicity in Batch Cultures and Empower Gene Function Discovery" has been accepted for publication in The Plant Cell, pending a final minor editorial review by journal staff. At this stage, your manuscript will be evaluated by a Science Editor with respect to its presentation of scientific content, compliance with journal policies, and presentation for a broad readership.

Final acceptance from Science Editor **Jan. 25, 2021**
